# Type material of Platyhelminthes (Monogenoidea) housed in the Helminthological Collection of the Oswaldo Cruz Institute/ FIOCRUZ (CHIOC), Rio de Janeiro, Brazil, from 1979 to 2016

**DOI:** 10.3897/zookeys.616.8481

**Published:** 2016-09-12

**Authors:** Daniela A. Lopes, Adriana Mainenti, Magda Sanches, Marcelo Knoff, Delir Corrêa Gomes

**Affiliations:** 1 Laboratório de Helmintos Parasitos de Vertebrados, Instituto Oswaldo Cruz, FIOCRUZ, Av. Brasil, 4365 Rio de Janeiro, RJ, Brazil

**Keywords:** Catalogue, helminths, holotype, monogenoids, paratype

## Abstract

A catalogue of type material of monogenoids deposited in the Helminthological Collection of the Oswaldo Cruz Institute, FIOCRUZ (CHIOC), between 1979 and 2016, is presented, given that the last list of types was produced in 1979. The monogenoid collection comprises type lots for 203 species, distributed across 14 families and 68 genera. Specific names are listed systematically, followed by type host, infection site, type locality, specimens with the collection numbers and references. The classification and the nomenclature of the species have been updated.

## Introduction

Natural history collections provide important documentary evidence of biodiversity and the information that they contain is very useful. In the case of helminthological collections, the specimens acquired have been a source not only for knowledge of helminth biodiversity, but also for parasitological studies on taxonomy, systematics and identification of causes of zoonoses. The century-old Helminthological Collection of the Oswaldo Cruz Institute, FIOCRUZ (CHIOC), Rio de Janeiro, Brazil, contains helminths that form part of the fauna of Brazil and other countries, from a wide range of hosts that were caught in different biomes. The samples are holotypes, paratypes and representative specimens of Platyhelminthes (Trematoda, Cestoda and Monogenoidea), Acanthocephala, Nematoda and other non-helminth phyla, such as Annelida and Arthropoda. Recently, a large number of species of Monogenoidea were deposited in the CHIOC, which reflects the increased number of taxonomists working on this group in Brazil.

## History and composition of the collection

The CHIOC celebrated its centenary in 2013. Its history is intertwined with that of the Laboratory of Helminth Parasites of Vertebrates of the Oswaldo Cruz Institute (LHPV–IOC), which began with the fieldwork of Gomes de Faria and his student Lauro Travassos (Faria and Travassos 1913). The Collection also received important contributions from other researchers at that time and, since then, it has increased in size through incorporation of private and institutional collections of the late nineteenth and early twentieth centuries (Noronha 2004, Noronha et al. 2004, 2009).

Today, the CHIOC holds around 38.400 samples of helminth parasites from South America and other continents. It is the biggest collection in Latin America and it is among the largest collections at a worldwide reference level (Rego 1982, Knoff et al. 2010), not only because of the number of types, but also because of the number of representative specimens (vouchers) that it contains. The collection has been constantly expanding through the large numbers of deposits made by Brazilian and foreign researchers. Some samples of arthropods (mainly pentastomids) and annelids (parasitic leeches) have also been accepted for inclusion in the collection, thus expanding its original nature of focusing on helminthological parasites of vertebrates, and resulting in a diversified parasitological collection.

All the material is organized according to the lots, which are numbered, recorded in an entry book, and digitized into a database. They are maintained in modern steel sliding closets and in a freezer that maintains some specimens and eggs of *Schistosoma mansoni* Sambon, 1907 (Trematoda) at -30 °C. Most of the information on the sample collections is available on the CHIOC web page (http://chioc.fiocruz.br/) and through the *speciesLink* network of the Environmental Information Reference Center (CRIA). Deposits and loans of specimens, permission for visits to examine samples *in loco*, loans of books and reprints, mini-courses and lectures focusing on the history of the CHIOC, and professional capacitation courses are included among the facilities of this Collection.

The first and only catalogue of the type material held in the CHIOC was published by Rego et al. (1979). They recorded 719 types (only holotypes or type series) of helminths, including 408 of nematodes, 227 of trematodes (Digenea and Monogenoidea), 52 of acanthocephalans, 28 of cestodes and four of pentastomids. Subsequent to this publication, the collection has grown substantially and the number of types has increased significantly as well. The purpose of this article is to inform the scientific community about the acquisitions of the CHIOC by publishing a catalogue of types deposited up to August 1, 2016. Because of the large number of types, we are starting with the species of Monogenoidea. We have followed the articles 73–75 of the Code (ICZN 1999).

## Material and methods

The specimens are stored in glass or plastic vials in 70% ethanol or as microscope slide preparations. All the material is available for consultation, but holotypes are not loaned. Unless otherwise stated, all type material is in good condition.

The catalogue is arranged taxonomically as subclasses, families, genera and species, under the original spelling and combinations. Subclasses are arranged following phylogenetic order. Families, genera and species are arranged alphabetically. The information on each entry is presented in the following format:

1. Original genus-species combination with author(s) and year of publication. Asterisk (*) denotes the type species of the genus.
2. Type host: species, author(s) and principal taxonomic group in brackets.
3. Infestation or infection site in the host.
4. Type locality: country, province or state, department, specific locality and coordinates (if available).
5. Primary type status: CHIOC catalogue number. The categories for the types used followed articles 73–75 of the Code (ICZN 1999).
6. Remark sections are inserted when necessary and include additional information about host, locality or status of the types.
7. References include publications in which the species was described and those that mention type specimens in the CHIOC.

The valid names adopted for parasitized hosts follow the most recent bibliography. Fish names are present in accordance with Froese and Pauly (2016) and turtle names, in accordance with Fritz and Havaš (2007, 2013). Mention of the monogenoids and host species in this list does not imply that the authors of the present report agree with their validity or taxonomy. Some monogenoids species catalogued have been synonymized and the comments about their taxonomy are in the Remarks sections. The higher classification of Monogenoidea follows Boeger and Kritsky (1993), and for families and genera follows specific references.

The following abbreviations are used in the text:

AHC SAMA
 Australian Helminthological Collection of the South Australian Museum, North Terrace, Adelaide, Australia 


BMNH
 British Museum of Natural History, Collection at the Department of Zoology, Natural History Museum, London, England 


CHIOC
 Helminthological Collection of the Oswaldo Cruz Institute, FIOCRUZ, Rio de Janeiro, Brazil 


CHMLP
 Helminthological Collection of the Museum of La Plata, La Plata, Buenos Aires, Argentina 


CNHE
 National Collection of Helminths, Institute of Biology, National Autonomous University of Mexico, Mexico City, Mexico 


HWML
 Harold W. Manter Laboratory, University of Nebraska State Museum, Lincoln, Nebraska, USA 


INPA
 National Institute for Amazon Research, Manaus, Amazonas, Brazil 


IPCAS/IPCR/ASCR
 Institute of Parasitology, Academy of Sciences of the Czech Republic, České Budějovice, Czech Republic 


MNHN
National Museum of Natural History, Paris, France 


MPEG
Invertebrate Collection of the Emílio Goeldi Museum, Belém, Pará, Brazil 


MPM
 Meguro Parasitological Museum, Meguro-ku, Tokyo, Japan 


MZUSP
 Helminthological Collection of the Zoology Museum, University of São Paulo, São Paulo, Brazil 


PC–IBPRAS
 Parasitological Collection of the Institute of Biology and Pedology, Russian Academy of Sciences, Vladivostok, Russia 


QM
Queensland Museum, Brisbane, Queensland, Australia 


UNICAMP
 Collection of the Natural History Museum of the University of Campinas, Campinas, São Paulo, Brazil 


USNM
 United States National Museum, Beltsville, Maryland, USA 


USNPC
 United States National Parasitological Collection, Beltsville, Maryland, USA 


ZIAC
 Zoological Institute of the Russian Academy of Sciences, Saint Petersburg, Russia 


## Results and discussion

This database and bibliographic survey presents the diversity of Monogenoidea types in CHIOC, from Brazil and other countries in the world, covering more than 35 years of parasitological studies. This catalogue is the second list of type species held in this Collection. The only previous checklist recorded only 11 type species of monogenoids, without considering paratypes (Rego et al. 1979). Now, there is a total of approximately 1100 primary types of Monogenoidea, represented by 203 species distributed across 14 families and 68 genera. Dactylogyridae is the most representative family, with 141 species, followed by Gyrodactylidae, with 29. These numbers illustrate the importance of the parasitological collection of the CHIOC, which is among the most significant in Latin America, together with other collections in Mexico and Argentina (Lamothe-Argumedo et al. 2010, Tablado and Tricárico 2010, Lunaschi et al. 2012).

It was observed that 93% of the type species of monogenoids catalogued in this Collection were parasites of bony fish. Only 12 species were parasites of cartilaginous fishes (Potamotrygonidae) and two monogenoids were parasites of turtles (Kinosternidae and Chelidae). All those samples were collected mainly in Brazil (95%), from all regions of this country. This situation could be a reflection of the large number of studies carried out on fish from Brazilian rivers and the Atlantic Ocean, and especially, of the increase in knowledge of monogenoids in Brazil that has been achieved since the late 1960s (Cohen et al. 2013).

### List of type species

#### Phylum Platyhelminthes Gegenbaur, 1859

##### Class Monogenoidea Bychowsky, 1937

###### Subclass Polyonchoinea Bychowsky, 1937

####### Capsalidae Baird, 1853

######## *Caballerocotyla* Price, 1960

######### Caballerocotyla lenti

Taxon classificationAnimaliaCapsalideaCapsalidae

Mogrovejo & Santos, 2002

########## Type host.

*Auxis thazard* (Lacépède, 1800) (Osteichthyes: Scombridae).

########## Infection site.

Gills.

########## Type locality.

Brazil, coastal zone of Rio de Janeiro State (22°55'S, 40°18'W).

########## Holotype.

CHIOC 34938.

########## Paratypes.

CHIOC 34939, 34940, 34941.

########## Remarks.

The species is referred to as *Caballerocotyla magronum* (Ishii, 1936) by Cohen et al. (2013). Gibson and Bray (2016) consider the species to be a synonym of *Capsala magronum* (Ishii, 1936).

########## References.

Mogrovejo and Santos (2002), Gibson and Bray (2016).

######### Caballerocotyla llewelyni

Taxon classificationAnimaliaCapsalideaCapsalidae

Kohn & Justo, 2006

########## Type host.

*Katsuwonus pelamis* (Linnaeus, 1758) (Osteichthyes: Scombridae).

########## Infection site.

Gills.

########## Type locality.

Brazil, coastal zone of Rio de Janeiro State (22°55'S, 40°18'W).

########## Holotype.

CHIOC 36611 a.

########## Paratype.

CHIOC 36611 b.

########## Remarks.

The species is referred to as *Caballerocotyla katsuwoni* (Ishii, 1936) by Cohen et al. (2013).Gibson and Bray (2016) consider the species to be a synonym of *Capsala katsuwoni* (Ishii, 1936).

########## References.

Kohn and Justo (2006), Gibson and Bray (2016).

######## *Encotylabe* Diesing, 1850

######### Encotyllabe souzalimae

Taxon classificationAnimaliaCapsalideaCapsalidae

Carvalho & Luque, 2012

########## Type host.

*Trichiurus lepturus* (Linnaeus, 1758) (Osteichthyes: Trichiuridae).

########## Infection site.

Gills.

########## Type locality.

Brazil, Rio de Janeiro State, Guanabara Bay (23°1'52"S, 43°11'56"W).

########## Holotype.

CHIOC 37382.

########## Paratypes.

CHIOC 37383, 37384, 37385.

########## Reference.

Carvalho and Luque (2012).

######## *Nasicola* Yamaguti, 1968

######### Nasicola brasiliensis

Taxon classificationAnimaliaCapsalideaCapsalidae

Kohn, Baptista-Farias, Santos & Gibson, 2004

########## Type host.

*Thunnus obesus* (Lowe, 1839) (Osteichthyes: Scombridae).

########## Infection site.

Nasal cavities.

########## Type locality.

Brazil, Rio de Janeiro State, off Cabo Frio (22°52'46"S, 42°01'07"W).

########## Holotype.

CHIOC 34924 a.

########## Paratypes.

CHIOC 34924 b, 34925 a–b, 34935.

########## Remarks.

Other paratypes deposited in the BMNH collection.

########## Reference.

Kohn et al. (2004).

######## *Sprostoniella* Bychowsky & Nagibina, 1967

######### Sprostoniella micrancyra

Taxon classificationAnimaliaCapsalideaCapsalidae

Cezar, Luque & Amato, 1999

########## Type host.

*Chaetodipterus faber* (Broussonet, 1782) (Osteichthyes: Ephippidae).

########## Infection site.

Gills.

########## Type locality.

Brazil, coastal zone of Rio de Janeiro State (21–23°S, 41–45°W).

########## Holotype.

CHIOC 34000 a.

########## Paratypes.

CHIOC 34000 b–d.

########## Remarks.

Other paratype deposited in CNHE.

########## Reference.

Cezar et al. (1999).

####### Dactylogyridae Bychowsky, 1933

######## *Ameloblastella* Kritsky, Mendoza-Franco & Scholz, 2000

######### Ameloblastella paranaensis

Taxon classificationAnimaliaDactylogyrideaDactylogyridae

(França, Isaac, Pavanelli & Takemoto, 2003) Mendoza-Franco & Scholz, 2009

########## Type host.

*Iheringichthys labrosus* (Lütken, 1874) (Osteichthyes: Pimelodidae).

########## Infection site.

Gills.

########## Type locality.

Brazil, upper Paraná River floodplain (22°43'S, 53°10'W).

########## Holotype.

CHIOC 34598 a.

########## Paratypes.

CHIOC 34598 b, 34588 a–d.

########## Remarks.

Material deposited as *Pseudovancleaveus paranaensis*. Mendoza-Franco and Scholz (2009) transferred *Pseudovancleaveus* to *Ameloblastella*.

########## References.

França et al. (2003), Mendoza-Franco and Scholz (2009), Monteiro et al. (2010a).

######## *Anacanthoroides* Kritsky & Thatcher, 1976

######### Anacanthoroides sanctifrancisci

Taxon classificationAnimaliaDactylogyrideaDactylogyridae

Monteiro & Brasil-Sato, 2014

########## Type host.

*Prochilodus argenteus* Spix & Agassiz, 1829 (Osteichthyes: Prochilodontidae).

########## Infection site.

Gill filaments.

########## Type locality.

Brazil, Minas Gerais State, São Francisco River near Três Marias Reservoir (18°12'32"S, 45°14'41"W).

########## Holotype.

CHIOC 37914.

########## Paratypes.

CHIOC 37915, 37916 a–b, 37917, 37918 a–c, 37919, 37920 a–b.

########## Reference.

Monteiro and Brasil-Sato (2014a).

######## *Anacanthorus* Mizelle & Price, 1965

######### Anacanthorus acuminatus

Taxon classificationAnimaliaDactylogyrideaDactylogyridae

Kristky, Boeger & Van Every, 1992

########## Type host.

*Triportheus angulatus* (Spix & Agassiz, 1829) (Osteichthyes: Triportheidae).

########## Infection site.

Gills.

########## Type locality.

Brazil, Amazonas State, Furo do Catalão near Manaus.

########## Paratype.

CHIOC 33369.

########## Remarks.

Specimen deposited in CHIOC collected from the type host/locality.Holotype deposited in the INPA collection. Other paratypes deposited in HWML. The CHIOC was cited in the original description as one of those collections of deposit, but its number was not informed there.

########## Reference.

Kritsky et al. (1992).

######### Anacanthorus adkruidenieri

Taxon classificationAnimaliaDactylogyrideaDactylogyridae

Monteiro, Cohen & Brasil-Sato, 2015

########## Type host.

*Salminus franciscanus* Lima & Britski, 2007 (Osteichthyes: Bryconidae).

########## Infection site.

Gills.

########## Type locality.

Brazil, Minas Gerais State, São Francisco River near Três Marias Reservoir (18°12'32"S, 45°15'41"W).

########## Holotype.

CHIOC 38018 a.

########## Paratypes.

CHIOC 38017 a–b, 38018 b–e, 38019 a–d, 38020 a–d

########## Reference.

Monteiro et al. (2015).

######### Anacanthorus alatus

Taxon classificationAnimaliaDactylogyrideaDactylogyridae

Kristky, Boeger & Van Every, 1992

########## Type host.

*Triportheus albus* Cope, 1872

########## Infection site.

Gills.

########## Type locality.

Brazil, Amazonas State, Furo do Catalão near Manaus.

########## Paratype.

CHIOC 33365.

########## Remarks.

Specimen deposited in CHIOC collected from the type host/locality. Holotype deposited in the INPA collection. Other paratypes deposited in HWML and USNM. The CHIOC was cited in the original description as one of those collections of deposit, but its number was not informed there.

########## Reference.

Kristky et al. (1992).

######### Anacanthorus amazonicus

Taxon classificationAnimaliaDactylogyrideaDactylogyridae

Van Every & Kristky, 1992

########## Type host.

*Serrasalmus rhombeus* (Linnaeus, 1766) (Osteichthyes: Serrasalmidae).

########## Infection site.

Gills.

########## Type locality.

Brazil, Amazonas State, Água Branca stream, Pitinga River.

########## Paratypes.

CHIOC 33389 a–b.

########## Remarks.

Specimens from CHIOC collected in the Uatamã River. Holotype deposited in the INPA collection. Other paratypes deposited in HWML and USNM. The CHIOC was cited in the original description as one of those collections of deposit, but its number was not informed there.

########## Reference.

Van Every and Kristky (1992).

######### Anacanthorus bellus

Taxon classificationAnimaliaDactylogyrideaDactylogyridae

Kristky, Boeger & Van Every, 1992

########## Type host.

*Triportheus albus*

########## Infection site.

Gills.

########## Type locality.

Brazil, Amazonas State, Furo do Catalão near Manaus.

########## Paratype.

CHIOC 33366.

########## Remarks.

Specimen deposited in CHIOC collected from the type host/locality. Holotype deposited in the INPA collection. Other paratypes deposited in HWML and USNM. The CHIOC was cited in the original description as one of those collections of deposit, but its number was not informed there.

########## Reference.

Kristky et al. (1992).

######### Anacanthorus bicuspidatus

Taxon classificationAnimaliaDactylogyrideaDactylogyridae

Cohen, Kohn & Boeger, 2012

########## Type host.

*Salminus brasiliensis* (Cuvier, 1816)

########## Infection site.

Gills.

########## Type locality.

Brazil, Paraná State, Paraná River, below and above of the reservoir of Itaipu Hydroelectric Power Station, in the locality of Guaíra (24°04'48"S, 54°15'21"W).

########## Holotype.

CHIOC 37595.

########## Paratypes.

CHIOC 37587, 37588, 37598 a–b, 37600, 37604, 37612, 37613, 37623, 37631, 37722, 37730.

########## Reference.

Cohen et al. (2012).

######### Anacanthorus brevicirrus

Taxon classificationAnimaliaDactylogyrideaDactylogyridae

Monteiro, Kritsky & Brasil-Sato, 2010

########## Type host.

*Brycon orthotaenia* (Günther, 1864) (Osteichthyes: Bryconidae).

########## Infection site.

Gills.

########## Type locality.

Brazil, Minas Gerais State, São Francisco River near Três Marias Reservoir (18°12'32"S, 45°15'41"W).

########## Holotype.

CHIOC 37333 a.

########## Paratypes.

CHIOC 37333 b–c, 37334.

########## Remarks.

Other paratypes deposited in the collections of INPA, IPCAS and USNM.

########## Reference.

Monteiro et al. (2010b).

######### Anacanthorus calophallus

Taxon classificationAnimaliaDactylogyrideaDactylogyridae

Kristky, Boeger & Van Every, 1992

########## Type host.

*Triportheus elongatus* (Günther, 1864)

########## Infection site.

Gills.

########## Type locality.

Brazil, Amazonas State, Manaus, Solimões River near Marchantaria Island.

########## Paratype.

CHIOC 33377.

########## Remarks.

Specimen deposited in CHIOC collected from the type host/locality. Holotype deposited in the INPA collection. Other paratypes deposited in HWML and USNM. The CHIOC was cited in the original description as one of those collections of deposit, but its number was not informed there.

########## Reference.

Kritsky et al. (1992).

######### Anacanthorus carinatus

Taxon classificationAnimaliaDactylogyrideaDactylogyridae

Kristky, Boeger & Van Every, 1992

########## Type host.

*Triportheus angulatus*

########## Infection site.

Gills.

########## Type locality.

Brazil, Amazonas State, Manaus, Bairro São Jorge.

########## Paratype.

CHIOC 33370.

########## Remarks.

Specimen deposited in CHIOC collected from the type host/locality. Holotype deposited in the INPA collection. Other paratypes deposited in HWML and USNM. The CHIOC was cited in the original description as one of those collections of deposit, but its number was not informed there.

########## Reference.

Kritsky et al. (1992).

######### Anacanthorus catoprioni

Taxon classificationAnimaliaDactylogyrideaDactylogyridae

Kristky, Boeger & Van Every, 1992

########## Type host.

*Catoprion mento* (Cuvier, 1819) (Osteichthyes: Serrasalmidae).

########## Infection site.

Gills.

########## Type locality.

Brazil, Amazonas State, Uatumã River, Balbina.

########## Paratype.

CHIOC 33383.

########## Remarks.

Specimen deposited in CHIOC collected from the type host/locality. Holotype deposited in the INPA collection. Other paratypes deposited in HWML and USNM. The CHIOC was cited in the original description as one of those collections of deposit, but its number was not informed there.

########## Reference.

Kritsky et al. (1992).

######### Anacanthorus chelophorus

Taxon classificationAnimaliaDactylogyrideaDactylogyridae

Kristky, Boeger & Van Every, 1992

########## Type host.

*Triportheus angulatus*

########## Infection site.

Gills.

########## Type locality.

Brazil, Amazonas State, Manaus, Bairro São Jorge.

########## Paratype.

CHIOC 33371.

########## Remarks.

Specimen deposited in CHIOC collected from the type host/locality. Holotype deposited in the INPA collection. Other paratypes deposited in HWML and USNM. The CHIOC was cited in the original description as one of those collections of deposit, but its number was not informed there.

########## Reference.

Kritsky et al. (1992).

######### Anacanthorus cinctus

Taxon classificationAnimaliaDactylogyrideaDactylogyridae

Van Every & Kristky, 1992

########## Type host.

*Pristobrycon striolatus* (Steindachner, 1908)

########## Infection site.

Gills.

########## Type locality.

Brazil, Amazonas State, Uatumã River, Samaumã Lake.

########## Paratypes.

CHIOC 33605 a–b.

########## Remarks.

Specimens deposited in CHIOC collected from the type host/locality. Holotype deposited in the INPA collection. Other paratypes deposited in HWML and USNM. The CHIOC was cited in the original description as one of those collections of deposit, but its number was not informed there.

########## Reference.

Van Every and Kritsky (1992).

######### Anacanthorus cladophallus

Taxon classificationAnimaliaDactylogyrideaDactylogyridae

Van Every & Kristky, 1992

########## Type host.

*Serrasalmus spilopleura* Kner, 1858

########## Infection site.

Gills.

########## Type locality.

Brazil, Amazonas State, Manaus, Solimões River near Marchantaria Island.

########## Paratypes.

CHIOC 33393 a–b.

########## Remarks.

Specimens deposited in CHIOC collected from the type host/locality. Holotype deposited in the INPA collection. Other paratypes deposited in HWML and USNM. The CHIOC was cited in the original description as one of those collections of deposit, but its number was not informed there.

########## Reference.

Van Every and Kritsky (1992).

######### Anacanthorus contortus

Taxon classificationAnimaliaDactylogyrideaDactylogyridae

Cohen, Kohn & Boeger, 2012

########## Type host.

*Salminus brasiliensis*

########## Infection site.

Gills.

########## Type locality.

Brazil, Paraná State, Paraná River, below and above of the reservoir of Itaipu Hydroelectric Power Station, Guaíra (24°04'48"S, 54°15'21"W).

########## Holotype.

CHIOC 37662 a.

########## Paratypes.

CHIOC 37583, 37601 a–c, 37605, 37615, 37620 a–b, 37621 a–b, 37626, 37632, 37660 a–c, 37662 b, 37705, 37706, 37707, 37714, 37716, 37718 a–b, 37721, 37724, 37727 a–b, 37729.

########## Reference.

Cohen et al. (2012).

######### Anacanthorus cornutus

Taxon classificationAnimaliaDactylogyrideaDactylogyridae

Kristky, Boeger & Van Every, 1992

########## Type host.

*Triportheus angulatus*

########## Infection site.

Gills.

########## Type locality.

Brazil, Amazonas State, Manaus, Bairro São Jorge.

########## Paratype.

CHIOC 33372.

########## Remarks.

Specimen deposited in CHIOC collected from the type host/locality. Holotype deposited in the INPA collection. Other paratypes deposited in HWML and USNM. The CHIOC was cited in the original description as one of those collections of deposit, but its number was not informed there.

########## Reference.

Kritsky et al. (1992).

######### Anacanthorus crytocaulus

Taxon classificationAnimaliaDactylogyrideaDactylogyridae

Van Every & Kristky, 1992

########## Type host.

*Pristobrycon striolatus*

########## Infection site.

Gills.

########## Type locality.

Brazil, Amazonas State, Uatumã River, Samaumã Lake.

########## Paratypes.

CHIOC 33606 a–b.

########## Remarks.

Specimens deposited in CHIOC collected from the type host/locality. Holotype deposited in the INPA collection. Other paratypes deposited in HWML and USNM. The CHIOC was cited in the original description as one of those collections of deposit, but its number was not informed there.

########## Reference.

Van Every and Kritsky (1992).

######### Anacanthorus daulometrus

Taxon classificationAnimaliaDactylogyrideaDactylogyridae

Cohen, Kohn & Boeger, 2012

########## Type host.

*Salminus brasiliensis*

########## Infection site.

Gills.

########## Type locality.

Brazil, Paraná State, Paraná River, below and above of the reservoir of Itaipu Hydroelectric Power Station, Guaíra (24°04'48"S, 54°15'21"W).

########## Holotype.

CHIOC 37681.

########## Paratypes.

CHIOC 37596, 37602, 37614, 37630, 37636, 37641, 37659, 37663, 37666 a–c, 37668, 37671, 37683, 37684, 37709 a–b, 37711 a–c, 37713 a–b, 37717, 37726, 37728.

########## Reference.

Cohen et al. (2012).

######### Anacanthorus dipelecinus

Taxon classificationAnimaliaDactylogyrideaDactylogyridae

Kristky, Boeger & Van Every, 1992

########## Type host.

*Roeboides myersii* Gill, 1870 (Osteichthyes: Characidae).

########## Infection site.

Gills.

########## Type locality.

Brazil, Amazonas State, Manaus, Solimões River near Marchantaria Island.

########## Paratype.

CHIOC 33600.

########## Remarks.

Specimen deposited in CHIOC collected from the type host/locality. Holotype deposited in the INPA collection. Other paratypes deposited in HWML and USNM. The CHIOC was cited in the original description as one of those collections of deposit, but its number was not informed there.

########## Reference.

Kritsky et al. (1992).

######### Anacanthorus douradensis

Taxon classificationAnimaliaDactylogyrideaDactylogyridae

Cohen, Kohn & Boeger, 2012

########## Type host.

*Salminus brasiliensis*

########## Infection site.

Gills.

########## Type locality.

Brazil, Paraná State, Paraná River, below and above of the reservoir of Itaipu Hydroelectric Power Station, Guaíra (24°04'48"S, 54°15'21"W).

########## Holotype.

CHIOC 37594.

########## Paratypes.

CHIOC 37584, 37599 a–b, 37610, 37611, 37616, 37654, 37679.

########## Reference.

Cohen et al. (2012).

######### Anacanthorus euryphallus

Taxon classificationAnimaliaDactylogyrideaDactylogyridae

Kristky, Boeger & Van Every, 1992

########## Type host.

*Triportheus angulatus*

########## Infection site.

Gills.

########## Type locality.

Brazil, Amazonas State, Furo do Catalão near Manaus.

########## Paratype.

CHIOC 33373.

########## Remarks.

Specimen deposited in CHIOC collected from the type host/locality. Holotype deposited in the INPA collection. Other paratypes deposited in HWML and USNM. The CHIOC was cited in the original description as one of those collections of deposit, but its number was not informed there.

########## Reference.

Kritsky et al. (1992).

######### Anacanthorus formosus

Taxon classificationAnimaliaDactylogyrideaDactylogyridae

Kristky, Boeger & Van Every, 1992

########## Type host.

*Triportheus elongatus*

########## Infection site.

Gills.

########## Type locality.

Brazil, Amazonas State, Furo do Catalão near Manaus.

########## Paratype.

CHIOC 33378.

########## Remarks.

Specimen deposited in CHIOC collected from the type host/locality. Holotype deposited in the INPA collection. Other paratypes deposited in HWML and USNM. The CHIOC was cited in the original description as one of those collections of deposit, but its number was not informed there.

########## Reference.

Kritsky et al. (1992).

######### Anacanthorus franciscanus

Taxon classificationAnimaliaDactylogyrideaDactylogyridae

Monteiro, Kritsky & Brasil-Sato, 2010

########## Type host.

*Brycon orthotaenia*

########## Infection site.

Gills.

########## Type locality.

Brazil, Minas Gerais State, São Francisco River near Três Marias Reservoir (18°12'32"S, 45°15'41"W).

########## Holotype.

CHIOC 37330.

########## Paratypes.

CHIOC 37331 a–e, 37332.

########## Remarks.

Other material deposited in the collections of INPA, IPCAS and USNM.

########## Reference.

Monteiro et al. (2010b).

######### Anacanthorus furculus

Taxon classificationAnimaliaDactylogyrideaDactylogyridae

Kristky, Boeger & Van Every, 1992

########## Type host.

*Triportheus elongatus*

########## Infection site.

Gills.

########## Type locality.

Brazil, Amazonas State, Solimões River near Manaus.

########## Paratype.

CHIOC 33379.

########## Remarks.

Specimen from CHIOC collected in the Manaus Fish market. Holotype deposited in the INPA collection. Other paratypes deposited in HWML and USNM. The CHIOC was cited in the original description as one of those collections of deposit, but its number was not informed there.

########## Reference.

Kritsky et al. (1992).

######### Anacanthorus glyptophallus

Taxon classificationAnimaliaDactylogyrideaDactylogyridae

Kristky, Boeger & Van Every, 1992

########## Type host.

*Triportheus angulatus*

########## Infection site.

Gills.

########## Type locality.

Brazil, Amazonas State, Manaus, Bairro São Jorge.

########## Paratype.

CHIOC 33374.

########## Remarks.

Specimen deposited in CHIOC collected from the type host/locality. Holotype deposited in the INPA collection. Other paratypes deposited in HWML and USNM. The CHIOC was cited in the original description as one of those collections of deposit, but its number was not informed there.

########## Reference.

Kritsky et al. (1992).

######### Anacanthorus gravihamulatus

Taxon classificationAnimaliaDactylogyrideaDactylogyridae

Van Every & Kristky, 1992

########## Type host.

*Serrasalmus rhombeus*

########## Infection site.

Gills.

########## Type locality.

Brazil, Amazonas State, Uatumã River, Água Branca stream, Pitinga River.

########## Paratypes.

CHIOC 33391 a–b.

########## Remarks.

Specimens deposited in CHIOC collected from the type host/locality. Holotype deposited in the INPA collection. Other paratypes deposited in HWML and USNM. The CHIOC was cited in the original description as one of those collections of deposit, but its number was not informed there.

########## Reference.

Van Every and Kristky (1992).

######### Anacanthorus jegui

Taxon classificationAnimaliaDactylogyrideaDactylogyridae

Van Every & Kristky, 1992

########## Type host.

*Serrasalmus rhombeus*

########## Infection site.

Gills.

########## Type locality.

Brazil, Amazonas State, Uatumã River, a tributary of Amazonas River.

########## Paratype.

CHIOC 33390.

########## Remarks.

Specimen from CHIOC collected in the Pitinga River, Água Branca stream (Amazonas State). Holotype deposited in the INPA collection. Other paratypes deposited in HWML and USNM. The CHIOC was cited in the original description as one of those collections of deposit, but its number was not informed there.

########## Reference.

Van Every and Kristky (1992).

######### Anacanthorus lasiophallus

Taxon classificationAnimaliaDactylogyrideaDactylogyridae

Van Every & Kristky, 1992

########## Type host.

*Pristobrycon striolatus*

########## Infection site.

Gills.

########## Type locality.

Brazil, Amazonas State, Água Branca stream, Pitinga River.

########## Paratypes.

CHIOC 33607 a–b.

########## Remarks.

Specimens from CHIOC collected in the Samaumã Lake, Uatumã River (Amazonas State). Holotype deposited in the INPA collection. Other paratypes deposited in HWML and USNM. The CHIOC was cited in the original description as one of those collections of deposit, but its number was not informed there.

########## Reference.

Van Every and Kristky (1992).

######### Anacanthorus lepyrophallus

Taxon classificationAnimaliaDactylogyrideaDactylogyridae

Kristky, Boeger & Van Every, 1992

########## Type host.

*Serrasalmus elongatus* Kner, 1858

########## Infection site.

Gills.

########## Type locality.

Brazil, Amazonas State, Negro River near Manaus.

########## Paratype.

CHIOC 33385.

########## Remarks.

Specimen deposited in CHIOC collected from the type host/locality. Holotype deposited in the INPA collection. Other paratypes deposited in HWML and USNM. The CHIOC was cited in the original description as one of those collections of deposit, but its number was not informed there.

########## Reference.

Kristky et al. (1992).

######### Anacanthorus mastigophallus

Taxon classificationAnimaliaDactylogyrideaDactylogyridae

Kristky, Boeger & Van Every, 1992

########## Type host.

*Pristobrycon eigenmanni* Norman, 1929

########## Infection site.

Gills.

########## Type locality.

Brazil, Amazonas State, Uatumã River, Nazaré.

########## Paratype.

CHIOC 33602.

########## Remarks.

Specimen from CHIOC collected in Santa Luzia, Uatumã River. Holotype deposited in the INPA collection. Other paratypes deposited in HWML and USNM. The CHIOC was cited in the original description as one of those collections of deposit, but its number was not informed there.

########## Reference.

Kristky et al. (1992).

######### Anacanthorus mesocondylus

Taxon classificationAnimaliaDactylogyrideaDactylogyridae

Van Every & Kristky, 1992

########## Type host.

*Serrasalmus elongatus*

########## Infection site.

Gills.

########## Type locality.

Brazil, Amazonas State, Manaus, Solimões River near Marchantaria Island.

########## Paratypes.

CHIOC 33384 a–b.

########## Remarks.

Specimens deposited in CHIOC collected from the type host/locality. Holotype deposited in the INPA collection. Other paratypes deposited in HWML and USNM. The CHIOC was cited in the original description as one of those collections of deposit, but its number was not informed there.

########## Reference.

Van Every and Kristky (1992).

######### Anacanthorus nanus

Taxon classificationAnimaliaDactylogyrideaDactylogyridae

Kristky, Boeger & Van Every, 1992

########## Type host.

*Triportheus angulatus*

########## Infection site.

Gills.

########## Type locality.

Brazil, Amazonas State, Manaus, Bairro São Jorge.

########## Paratype.

CHIOC 33375.

########## Remarks.

Specimen deposited in CHIOC collected from the type host/locality. Holotype deposited in the INPA collection. Other paratypes deposited in HWML and USNM. The CHIOC was cited in the original description as one of those collections of deposit, but its number was not informed there.

########## Reference.

Kristky et al. (1992).

######### Anacanthorus palamophallus

Taxon classificationAnimaliaDactylogyrideaDactylogyridae

Kristky, Boeger & Van Every, 1992

########## Type host.

*Pristobrycon eigenmanni*

########## Infection site.

Gills.

########## Type locality.

Brazil, Amazonas State, Uatumã River, Nazaré.

########## Paratype.

CHIOC 33603.

########## Remarks.

Specimen deposited in CHIOC collected from the type host/locality. Holotype deposited in the INPA collection. Other paratypes deposited in HWML and USNM. The CHIOC was cited in the original description as one of those collections of deposit, but its number was not informed there.

########## Reference.

Kristky et al. (1992).

######### Anacanthorus paradouradensis

Taxon classificationAnimaliaDactylogyrideaDactylogyridae

Monteiro, Cohen & Brasil-Sato, 2015

########## Type host.

*Salminus franciscanus*

########## Infection site.

Gills.

########## Type locality.

Brazil, Minas Gerais State, São Francisco River near Três Marias Reservoir (18°12'32"S, 45°15'41"W).

########## Holotype.

CHIOC 38023 a.

########## Paratypes.

CHIOC 38021 a–d, 38022 a–c, 38023 b–d, 38024 a–d.

########## Reference.

Monteiro et al. (2015).

######### Anacanthorus parakruidenieri

Taxon classificationAnimaliaDactylogyrideaDactylogyridae

Cohen, Kohn & Boeger, 2012

########## Type host.

*Salminus brasiliensis*

########## Infection site.

Gills.

########## Type locality.

Brazil, Paraná State, Paraná River, below and above of the reservoir of Itaipu Hydroelectric Power Station, Guaíra (24°04'48"S, 54°15'21"W).

########## Holotype.

CHIOC 37656.

########## Paratypes.

CHIOC 37603 a–b, 37617, 37624, 37633 a–b, 37639, 37661 a–b, 37697.

########## Reference.

Cohen et al. (2012).

######### Anacanthorus paraspathulatus

Taxon classificationAnimaliaDactylogyrideaDactylogyridae

Kristky, Boeger & Van Every, 1992

########## Type host.

*Mylossoma duriventre* (Cuvier, 1818) [= *Mylossoma duriventris*] (Osteichthyes: Serrasalmidae).

########## Infection site.

Gills.

########## Type locality.

Brazil, Amazonas State, Manaus, Solimões River near Marchantaria Island.

########## Paratype.

CHIOC 33396.

########## Remarks.

Specimen deposited in CHIOC collected from the type host/locality. Holotype deposited in the INPA collection. Other paratypes deposited in HWML and USNM. The CHIOC was cited in the original description as one of those collections of deposit, but its number was not informed there.

########## Reference.

Kristky et al. (1992).

######### Anacanthorus pedanophallus

Taxon classificationAnimaliaDactylogyrideaDactylogyridae

Kristky, Boeger & Van Every, 1992

########## Type host.

*Myleus rubripinnis* (Müller & Troschel, 1844) [= *Myleus rubripinnus*] (Osteichthyes: Serrasalmidae).

########## Infection site.

Gills.

########## Type locality.

Brazil, Amazonas State, Uatumã River, Nazaré.

########## Paratype.

CHIOC 33397.

########## Remarks.

Specimen deposited in CHIOC collected from the type host/locality. Holotype deposited in the INPA collection. Other paratypes deposited in HWML and USNM. The CHIOC was cited in the original description as one of those collections of deposit, but its number was not informed there.

########## Reference.

Kristky et al. (1992).

######### Anacanthorus pelorophallus

Taxon classificationAnimaliaDactylogyrideaDactylogyridae

Kristky, Boeger & Van Every, 1992

########## Type host.

*Triportheus elongatus*

########## Infection site.

Gills.

########## Type locality.

Brazil, Amazonas State, Manaus, Solimões River near Marchantaria Island.

########## Paratype.

CHIOC 33380.

########## Remarks.

Specimen from CHIOC collected in the Manaus Fish market. Holotype deposited in the INPA collection. Other paratypes deposited in HWML and USNM. The CHIOC was cited in the original description as one of those collections of deposit, but its number was not informed there.

########## Reference.

Kristky et al. (1992).

######### Anacanthorus penilabiatus

Taxon classificationAnimaliaDactylogyrideaDactylogyridae

Boeger, Husak & Martins, 1995

########## Type host.

*Piaractus mesopotamicus* (Holmberg, 1887) (Osteichthyes: Serrasalmidae).

########## Infection site.

Gills.

########## Type locality.

Brazil, São Paulo State, Jaboticabal, Aquaculture Center of the Paulista State University.

########## Holotype.

CHIOC 33268 a.

########## Paratypes.

CHIOC 33268 b–j.

########## Remarks.

Other paratypes deposited in the collections of HWML and USNM.

########## Reference.

Boeger et al. (1995b).

######### Anacanthorus periphallus

Taxon classificationAnimaliaDactylogyrideaDactylogyridae

Kristky, Boeger & Van Every, 1992

########## Type host.

*Serrasalmus* sp.

########## Infection site.

Gills.

########## Type locality.

Brazil, Amazonas State, Furo do Catalão near Manaus.

########## Paratype.

CHIOC 33395.

########## Remarks.

Specimen deposited in CHIOC collected from the type host/locality. Holotype deposited in the INPA collection. Other paratypes deposited in HWML and USNM. The CHIOC was cited in the original description as one of those collections of deposit, but its number was not informed there.

########## Reference.

Kristky et al. (1992).

######### Anacanthorus pithophallus

Taxon classificationAnimaliaDactylogyrideaDactylogyridae

Kristky, Boeger & Van Every, 1992

########## Type host.

*Triportheus angulatus*

########## Infection site.

Gills.

########## Type locality.

Brazil, Amazonas State, Manaus, Bairro São Jorge.

########## Paratype.

CHIOC 33376.

########## Remarks.

Specimen deposited in CHIOC collected from the type host/locality. Holotype deposited in the INPA collection. Other paratypes deposited in HWML and USNM. The CHIOC was cited in the original description as one of those collections of deposit, but its number was not informed there.

########## Reference.

Kristky et al. (1992).

######### Anacanthorus prodigiosus

Taxon classificationAnimaliaDactylogyrideaDactylogyridae

Van Every & Kristky, 1992

########## Type host.

*Serrasalmus elongatus*

########## Infection site.

Gills.

########## Type locality.

Brazil, Amazonas State, Negro River near Manaus.

########## Paratypes.

CHIOC 33386 a–b.

########## Remarks.

Specimen deposited in CHIOC collected from the type host/locality. Holotype deposited in the INPA collection. Other paratypes deposited in HWML and USNM. The CHIOC was cited in the original description as one of those collections of deposit, but its number was not informed there.

########## Reference.

Van Every and Kristky (1992).

######### Anacanthorus quinqueramus

Taxon classificationAnimaliaDactylogyrideaDactylogyridae

Kristky, Boeger & Van Every, 1992

########## Type host.

*Triportheus albus*

########## Infection site.

Gills.

########## Type locality.

Brazil, Amazonas State, Furo do Catalão near Manaus.

########## Paratype.

CHIOC 33367.

########## Remarks.

Specimen deposited in CHIOC collected from the type host/locality. Holotype deposited in the INPA collection. Other paratypes deposited in HWML and USNM. The CHIOC was cited in the original description as one of those collections of deposit, but its number was not informed there.

########## Reference.

Kristky et al. (1992).

######### Anacanthorus ramosissimus

Taxon classificationAnimaliaDactylogyrideaDactylogyridae

Van Every Boeger & Kristky, 1992

########## Type host.

*Serrasalmus elongatus*

########## Infection site.

Gills.

########## Type locality.

Brazil, Amazonas State, Manaus, Solimões River near Marchantaria Island.

########## Paratypes.

CHIOC 33387 a–b.

########## Remarks.

Specimens deposited in CHIOC collected from the type host/locality. Holotype deposited in the INPA collection. Other paratypes deposited in HWML and USNM. The CHIOC was cited in the original description as one of those collections of deposit, but its number was not informed there.

########## Reference.

Van Every and Kristky (1992).

######### Anacanthorus ramulosus

Taxon classificationAnimaliaDactylogyrideaDactylogyridae

Kristky, Boeger & Van Every, 1992

########## Type host.

*Triportheus albus*

########## Infection site.

Gills.

########## Type locality.

Brazil, Amazonas State, Furo do Catalão near Manaus.

########## Paratype.

CHIOC 33368.

########## Remarks.

Specimen deposited in CHIOC collected from the type host/locality. Holotype deposited in the INPA collection. Other paratypes deposited in HWML and USNM. The CHIOC was cited in the original description as one of those collections of deposit, but its number was not informed there.

########## Reference.

Kristky et al. (1992).

######### Anacanthorus scapanus

Taxon classificationAnimaliaDactylogyrideaDactylogyridae

Van Every & Kristky, 1992

########## Type host.

*Serrasalmus spilopleura*

########## Infection site.

Gills.

########## Type locality.

Brazil, Amazonas State, Manaus, Solimões River near Marchantaria Island.

########## Paratype.

CHIOC 33394.

########## Remarks.

Specimen deposited in CHIOC collected from the type host/locality. Holotype deposited in the INPA collection. Other paratypes deposited in HWML and USNM. The CHIOC was cited in the original description as one of those collections of deposit, but its number was not informed there.

########## Reference.

Van Every and Kristky (1992).

######### Anacanthorus sciponophallus

Taxon classificationAnimaliaDactylogyrideaDactylogyridae

Van Every & Kristky, 1992

########## Type host.

*Serrasalmus elongatus*

########## Infection site.

Gills.

########## Type locality.

Brazil, Amazonas State, Manaus, Solimões River near Marchantaria Island.

########## Paratypes.

CHIOC 33388 a–b.

########## Remarks.

Specimens deposited in CHIOC collected from the type host/locality. Holotype deposited in the INPA collection. Other paratypes deposited in HWML and USNM. The CHIOC was cited in the original description as one of those collections of deposit, but its number was not informed there.

########## Reference.

Van Every and Kristky (1992).

######### Anacanthorus serrasalmi

Taxon classificationAnimaliaDactylogyrideaDactylogyridae

Van Every & Kristky, 1992

########## Type host.

*Serrasalmus rhombeus*

########## Infection site.

Gills.

########## Type locality.

Brazil, Amazonas State, Água Branca stream, Pitinga River.

########## Paratypes.

CHIOC 33392 a–b.

########## Remarks.

Specimens from CHIOC collected in the Uatumã River (Amazonas State). Holotype deposited in the INPA collection. Other paratypes deposited in HWML and USNM. The CHIOC was cited in the original description as one of those collections of deposit, but its number was not informed there.

########## Reference.

Van Every and Kristky (1992).

######### Anacanthorus spinatus

Taxon classificationAnimaliaDactylogyrideaDactylogyridae

Kristky, Boeger & Van Every, 1992

########## Type host.

*Myleus rubripinnis*

########## Infection site.

Gills.

########## Type locality.

Brazil, Amazonas State, Uatumã River, Nazaré.

########## Paratype.

CHIOC 33398.

########## Remarks.

Specimen deposited in CHIOC collected from the type host/locality. Holotype deposited in the INPA collection. Other paratypes deposited in HWML and USNM. The CHIOC was cited in the original description as one of those collections of deposit, but its number was not informed there.

########## Reference.

Kristky et al. (1992).

######### Anacanthorus stachophallus

Taxon classificationAnimaliaDactylogyrideaDactylogyridae

Kristky, Boeger & Van Every, 1992

########## Type host.

*Pygocentrus nattereri* Kner, 1858 (Osteichthyes: Serrasalmidae).

########## Infection site.

Gills.

########## Type locality.

Brazil, Amazonas State, Manaus, Solimões River near Marchantaria Island.

########## Paratype.

CHIOC 33601.

########## Remarks.

Specimen deposited in CHIOC collected from the type host/locality. Holotype deposited in the INPA collection. Other paratypes deposited in HWML and USNM. The CHIOC was cited in the original description as one of those collections of deposit, but its number was not informed there.

########## Reference.

Kristky et al. (1992).

######### Anacanthorus stagmophallus

Taxon classificationAnimaliaDactylogyrideaDactylogyridae

Kristky, Boeger & Van Every, 1992

########## Type host.

*Myleus rubripinnis*

########## Infection site.

Gills.

########## Type locality.

Brazil, Amazonas State, Uatumã River, Nazaré.

########## Paratype.

CHIOC 33399.

########## Remarks.

Specimen deposited in CHIOC collected from the type host/locality. Holotype deposited in the INPA collection. Other paratypes deposited in HWML and USNM. The CHIOC was cited in the original description as one of those collections of deposit, but its number was not informed there.

########## Reference.

Kristky et al. (1992).

######### Anacanthorus strongylophallus

Taxon classificationAnimaliaDactylogyrideaDactylogyridae

Kristky, Boeger & Van Every, 1992

########## Type host.

*Triportheus elongatus*

########## Infection site.

Gills.

########## Type locality.

Brazil, Amazonas State, Manaus, Solimões River near Marchantaria Island.

########## Paratype.

CHIOC 33381.

########## Remarks.

Specimen deposited in CHIOC collected from the type host/locality. Holotype deposited in the INPA collection. Other paratypes deposited in HWML and USNM. The CHIOC was cited in the original description as one of those collections of deposit, but its number was not informed there.

########## Reference.

Kristky et al. (1992).

######### Anacanthorus toledoensis

Taxon classificationAnimaliaDactylogyrideaDactylogyridae

Leão, São Clemente & Cohen, 2015

########## Type host.

*Piaractus mesopotamicus*

########## Infection site.

Gills.

########## Type locality.

Brazil, Paraná State, Paraná River, in the locality of Toledo (24°44'29.0"S, 53°44'51.2"W).

########## Holotype.

CHIOC 37905 a.

########## Paratypes.

CHIOC 37905 b–c.

########## Remarks.

Other paratypes deposited in the INPA collection.

########## Reference.

Leão et al. (2015).

######### Anacanthorus tricornis

Taxon classificationAnimaliaDactylogyrideaDactylogyridae

Kristky, Boeger & Van Every, 1992

########## Type host.

*Triportheus elongatus*

########## Infection site.

Gills.

########## Type locality.

Brazil, Amazonas State, Manaus, Solimões River near Marchantaria Island.

########## Paratype.

CHIOC 33382.

########## Remarks.

Specimen deposited in CHIOC collected from the type host/locality. Holotype deposited in the INPA collection. Other paratypes deposited in HWML and USNM. The CHIOC was cited in the original description as one of those collections of deposit, but its number was not informed there.

########## Reference.

Kristky et al. (1992).

######### Anacanthorus xaniophallus

Taxon classificationAnimaliaDactylogyrideaDactylogyridae

Kristky, Boeger & Van Every, 1992

########## Type host.

*Pristobrycon eigenmanni*

########## Infection site.

Gills.

########## Type locality.

Brazil, Amazonas State, Uatumã River, Nazaré.

########## Paratypes.

CHIOC 33604 a–c.

########## Remarks.

Specimens deposited in CHIOC collected from the type host/locality. Holotype deposited in the INPA collection. Other paratypes deposited in HWML and USNM. The CHIOC was cited in the original description as one of those collections of deposit, but its number was not informed there.

########## Reference.

Kristky et al. (1992).

######## *Annulotrematoides* Kritsky & Boeger, 1995

######### * Annulotrematoides amazonicus

Taxon classificationAnimaliaDactylogyrideaDactylogyridae

Kritsky & Boeger, 1995

########## Type host.

*Psectrogaster rutiloides* (Kner, 1858) (Osteichthyes: Curimatidae).

########## Infection site.

Gills.

########## Type locality.

Brazil, Amazonas State, Manaus, Furo do Catalão.

########## Holotype.

CHIOC 33364.

########## Remarks.

Collection number referred to as CHIOC “33664” in the original description due to a mistake. Paratypes deposited in HWML and USNPC.

########## Reference.

Kritsky and Boeger (1995).

######### Annulotrematoides bryconi

Taxon classificationAnimaliaDactylogyrideaDactylogyridae

Cuglianna, Cordeiro & Luque, 2003

########## Type host.

*Brycon cephalus* (Günther, 1869)

########## Infection site.

Gills.

########## Type locality.

Brazil, São Paulo State, ponds in Pirassununga.

########## Holotype.

CHIOC 36276.

########## Paratypes.

CHIOC 36272, 36273 a–b, 36274 a–b, 36275.

########## Remarks.

Four paratypes deposited in UNICAMP.

########## Reference.

Cuglianna et al. (2003).

######### Annulotrematoides glossophallus

Taxon classificationAnimaliaDactylogyrideaDactylogyridae

Cohen, Kohn & Boeger, 2012

########## Type host.

*Salminus brasiliensis*

########## Infection site.

Gills.

########## Type locality.

Brazil, Paraná State, Paraná River below and above of the reservoir of Itaipu Hydroelectric Power Station, Guaíra (24°04'48"S, 54°15'21"W).

########## Holotype.

CHIOC 37607.

########## Paratypes.

CHIOC 37586, 37618, 37625, 37627, 37629, 37642, 37657 a–b, 37669, 37682.

########## Reference.

Cohen et al. (2012).

######### Annulotrematoides parisellei

Taxon classificationAnimaliaDactylogyrideaDactylogyridae

Cohen, Kohn & Boeger, 2012

########## Type host.

*Salminus brasiliensis*

########## Infection site.

Gills.

########## Type locality.

Brazil, Paraná State, Paraná River below and above of the reservoir of Itaipu Hydroelectric Power Station, Guaíra (24°04'48"S, 54°15'21"W).

########## Holotype.

CHIOC 37689.

########## Paratypes.

CHIOC 37585, 37589, 37590, 37591, 37592, 37593, 37597, 37608, 37619, 37622 a–b, 37634, 37643, 37648, 37649 a–b, 37652, 37658, 37664 a–b, 37667, 37676, 37677, 37680, 37685, 37686, 37692 a–b, 37694, 37698 a–b, 37701, 37715, 37725.

########## Reference.

Cohen et al. (2012).

######## *Apedunculata* Cuglianna, Cordeiro & Luque, 2009

######### * Apedunculata discoidea

Taxon classificationAnimaliaDactylogyrideaDactylogyridae

Cuglianna, Cordeiro & Luque, 2009

########## Type host.

*Prochilodus lineatus* (Valenciennes, 1837)

########## Infection site.

Gills.

########## Type locality.

Brazil, São Paulo State, ponds in Pirassununga.

########## Holotype.

CHIOC 36904.

########## Paratypes.

CHIOC 36905, 36906, 36907, 36908.

########## Reference.

Cuglianna et al. (2009).

######## *Aphanoblastella* Kritsky, Mendoza-Franco & Scholz, 2000

######### Aphanoblastella juizforense

Taxon classificationAnimaliaDactylogyrideaDactylogyridae

Carvalho, Tavares & Luque, 2009

########## Type host.

*Rhamdia quelen* (Quoy & Gaimard, 1824) (Osteichthyes: Heptapteridae).

########## Infection site.

Gills.

########## Type locality.

Brazil, Minas Gerais State, Juiz de Fora, Paraibuna River (21°41'20"S, 43°20'40"W).

########## Holotype.

CHIOC 37175 a.

########## Paratypes.

CHIOC 37175 b–e.

########## Reference.

Carvalho et al. (2009).

######## *Cacatuocotyle* Boeger, Domingues & Kritsky, 1997

######### Cacatuocotyle guaibensis

Taxon classificationAnimaliaDactylogyrideaDactylogyridae

Gallas, Calegaro-Marques & Amato, 2014

########## Type host.

Astyanax aff. fasciatus (Cuvier, 1819) (Osteichthyes: Characidae).

########## Infection site.

External surface.

########## Type locality.

Brazil, Rio Grande do Sul State, Guaíba Lake, Barra do Ribeiro (30°17'11"S, 51°18'01"W).

########## Holotype.

CHIOC 37965 a.

########## Paratypes.

CHIOC 37965 b–c.

########## Reference.

Gallas et al. (2014).

######### * Cacatuocotyle paranaensis

Taxon classificationAnimaliaDactylogyrideaDactylogyridae

Boeger, Domingues & Kritsky, 1997

########## Type host.

*Characidium lanei* Travassos, 1967 (Osteichthyes: Crenuchidae).

########## Infection site.

Gills.

########## Type locality.

Brazil, Paraná State, Antonina, Cacatu River.

########## Holotype.

CHIOC 33682 a.

########## Paratypes.

CHIOC 33683 a–b, 33684 a–e, 33685 a–d.

########## Remarks.

All paratypes were collected from *Cacatuocotyle pterostictum* Gomes, 1947, except CHIOC 33685 b, which was collected from the type host. Other paratypes deposited in HWML and USNPC.

########## Reference.

Boeger et al. (1997).

######## *Chauhanellus* Bychowsky & Nagibina, 1969

######### Chauhanellus boegeri

Taxon classificationAnimaliaDactylogyrideaDactylogyridae

Domingues & Fehlauer, 2006

########## Type host.

*Genidens barbus* (Lacepède, 1803) (Osteichthyes: Ariidae).

########## Infection site.

Gills.

########## Type locality.

Brazil, Paraná State, Guaratuba, Guaratuba Bay (25°52'16.73"S, 48°39'07.32"W).

########## Paratypes.

CHIOC 36821 a–e.

########## Remarks.

Specimens deposited in CHIOC collected from the type host/locality. Holotype deposited in MZUSP. Other paratypes deposited in MZUSP and USNPC.

########## Reference.

Domingues and Fehlauer (2006).

######### Chauhanellus hamatopeduncularoideum

Taxon classificationAnimaliaDactylogyrideaDactylogyridae

Domingues, Soares & Watanabe, 2016

########## Type host.

*Amphiarius rugispinis* (Valenciennes, 1840) (Osteichthyes: Ariidae).

########## Infection site.

Secondary lamellae of the gills.

########## Type locality.

Brazil, Pará State, Bragança, Fishing village of Ajuruteua (0°49'31"N, 46°36'29"W).

########## Holotype.

CHIOC 38240 a.

########## Paratypes.

CHIOC 38240 b–h.

########## Remarks.

Other paratypes deposited in the collections of INPA and MPEG.

########## Reference.

Domingues et al. (2016).

######### Chauhanellus hypenocleithrum

Taxon classificationAnimaliaDactylogyrideaDactylogyridae

Domingues, Soares & Watanabe, 2016

########## Type host.

*Sciades proops* (Valenciennes, 1840) (Osteichthyes: Ariidae).

########## Infection site.

Secondary lamellae of the gills.

########## Type locality.

Brazil, Pará State, Bragança, Fishing village of Ajuruteua (0°49'31"N, 46°36'29"W).

########## Holotype.

CHIOC 38247.

########## Paratypes.

CHIOC 38248 a–b, 38249 a–b, 38250 a–b.

########## Remarks.

Other paratypes deposited in the collections of INPA and MPEG.

########## Reference.

Domingues et al. (2016).

######### Chauhanellus neotropicalis

Taxon classificationAnimaliaDactylogyrideaDactylogyridae

Domingues & Fehlauer, 2006

########## Type host.

*Aspistor luniscutis* (Valenciennes, 1840) (Osteichthyes: Ariidae).

########## Infection site.

Gills.

########## Type locality.

Brazil, Paraná State, Paranaguá, Fish market.

########## Paratypes.

CHIOC 36823 a–b.

########## Remarks.

Holotype deposited in MZUSP. Other paratypes deposited in MZUSP and USNPC.

########## Reference.

Domingues and Fehlauer (2006).

######### Chauhanellus susamlimae

Taxon classificationAnimaliaDactylogyrideaDactylogyridae

Domingues, Soares & Watanabe, 2016

########## Type host.

*Sciades herzbergii* (Bloch, 1794)

########## Infection site.

Secondary lamellae of the gills.

########## Type locality.

Brazil, Pará State, Bragança, Fishing village of Ajuruteua (0°49'31"N, 46°36'29"W).

########## Holotype.

CHIOC 38251 a.

########## Paratypes.

CHIOC 38251 b–h.

########## Remarks.

Other paratypes deposited in the collections of INPA and MPEG.

########## Reference.

Domingues et al. (2016).

######### Chauhanellus velum

Taxon classificationAnimaliaDactylogyrideaDactylogyridae

Domingues, Soares & Watanabe, 2016

########## Type host.

*Sciades couma* (Valenciennes, 1840)

########## Infection site.

Internal borders of the primary lamellae of the gills.

########## Type locality.

Brazil, Pará State, Bragança, Fish market.

########## Holotype.

CHIOC 38261.

########## Remarks.

Paratype deposited in the INPA collection.

########## Reference.

Domingues et al. (2016).

######## *Demidospermus* Suriano, 1983

######### Demidospermus araguaiaensis

Taxon classificationAnimaliaDactylogyrideaDactylogyridae

Cepeda & Luque, 2010

########## Type host.

*Brachyplatystoma filamentosum* (Lichtenstein, 1819) (Osteichthyes: Pimelodidae).

########## Infection site.

Gill lamellae.

########## Type locality.

Brazil, Mato Grosso State, Araguaia River (13°23'07.3"S, 50°39'58.1"W).

########## Holotype.

CHIOC 37326.

########## Paratype.

CHIOC 37327.

########## Remarks.

Other paratype deposited in the INPA collection.

########## Reference.

Cepeda and Luque (2010).

######### Demidospermus brachyplatystomae

Taxon classificationAnimaliaDactylogyrideaDactylogyridae

Cepeda & Luque, 2010

########## Type host.

*Brachyplatystoma filamentosum*

########## Infection site.

Gill lamellae.

########## Type locality.

Brazil, Mato Grosso State, Araguaia River (13°23'07.3"S, 50°39'58.1"W).

########## Holotype.

CHIOC 37320.

########## Paratypes.

CHIOC 37321 a–b, 37322 a–d.

########## Remarks.

Other paratypes deposited in the INPA collection.

########## Reference.

Cepeda and Luque (2010).

######### Demidospermus ceccarellii

Taxon classificationAnimaliaDactylogyrideaDactylogyridae

Cepeda & Luque, 2010

########## Type host.

*Brachyplatystoma filamentosum*

########## Infection site.

Gill lamellae.

########## Type locality.

Brazil, Mato Grosso State, Araguaia River (13°23'07.3"S, 50°39'58.1"W).

########## Holotype.

CHIOC 37323.

########## Paratypes.

CHIOC 37324 a–d, 37325 a–e.

########## Remarks.

Other paratypes deposited in the INPA collection.

########## Reference.

Cepeda and Luque (2010).

######### Demidospermus labrosi

Taxon classificationAnimaliaDactylogyrideaDactylogyridae

França, Isaac, Pavanelli & Takemoto, 2003

########## Type host.

*Iheringichthys labrosus*

########## Infection site.

Gills.

########## Type locality.

Brazil, upper Paraná River floodplain (22°43'S, 53°10'W).

########## Holotype.

CHIOC 34594 a.

########## Paratypes.

CHIOC 34587 a–c, 34594 b–c, 34595, 34596, 34597.

########## Remarks.

Some authors considered the species to be a synonym of *Demidospermus cornicinus* Kritsky & Gutiérrez, 1998 (Cohen and Kohn 2008a, Monteiro et al. 2010a). Takemoto et al. (2009) referred to it as *Demidospermus labrosi*.

########## References.

França et al. (2003), Cohen and Kohn (2008a), Takemoto et al. (2009), Monteiro et al. (2010a).

######### Demidospermus mandi

Taxon classificationAnimaliaDactylogyrideaDactylogyridae

França, Isaac, Pavanelli & Takemoto, 2003

########## Type host.

*Iheringichthys labrosus*

########## Infection site.

Gills.

########## Type locality.

Brazil, upper Paraná River floodplain (22°43'S, 53°10'W).

########## Holotype.

CHIOC 34586 a.

########## Paratypes.

CHIOC 34586 b, 34589, 34590, 34591, 34592, 34593 a–b.

########## Remarks.

Some authors considered the species to be a synonym of *Demidospermus leptosynophallus* Kritsky & Gutiérrez, 1998 (Cohen and Kohn 2008a, Monteiro et al. 2010a). Takemoto et al. (2009) referred to it as *Demidospermus mandi*.

########## References.

França et al. (2003), Cohen and Kohn (2008a), Takemoto et al. (2009), Monteiro et al. (2010a).

######### Demidospermus osteomystax

Taxon classificationAnimaliaDactylogyrideaDactylogyridae

Tavernari, Takemoto, Lacerda & Pavanelli, 2010

########## Type host.

*Auchenipterus osteomystax* Miranda-Ribeiro, 1918 (Osteichthyes: Auchenipteridae).

########## Infection site.

Gills.

########## Type locality.

Brazil, upper Paraná River floodplain (22°50'–22°70'S, 53°15'–53°40'W).

########## Holotype.

CHIOC 37252 a.

########## Paratypes.

CHIOC 37252 b, 37253, 37254.

########## Reference.

Tavernari et al. (2010).

######### Demidospermus paranaensis

Taxon classificationAnimaliaDactylogyrideaDactylogyridae

Ferrari-Hoeinghaus, Bellay, Takemoto & Pavanelli, 2010

########## Type host.

*Loricariichthys platymetopon* Isbrücker & Nijssen, 1979 (Osteichthyes: Loricariidae).

########## Infection site.

Gill filaments.

########## Type locality.

Brazil, upper Paraná River floodplain (22°43'S, 53°10'W).

########## Holotype.

CHIOC 37255 a.

########## Paratypes.

CHIOC 37255 b–e.

########## Reference.

Ferrari-Hoeinghaus et al. (2010).

######## *Euryhaliotrema* Kritsky & Boeger, 2002

######### Euryhaliotrema dontykoleos

Taxon classificationAnimaliaDactylogyrideaDactylogyridae

Fehlauer & Boeger, 2005

########## Type host.

*Pachyurus junki* Soares & Casatti, 2000 (Osteichthyes: Sciaenidae).

########## Infection site.

Gills.

########## Type locality.

Brazil, Tocantins State, Santa Rosa, São Valério River (11°21'57"S, 48°28'35"W)

########## Paratypes.

CHIOC 36497, 36498, 36499 a–c, 36500.

########## Remarks.

Paratypes collected in different localities of the Tocantins River (Tocantins State): CHIOC 36498 (Peixe, 11°46'19"S, 48°27'26"W), 36499 a–c (Ipueiras, 11°18'50"S, 48°27'26"W) and 36500 (Porto Nacional, 10°41'45"S, 48°21'43"W). Holotype deposited in MZUSP. Other paratypes deposited in MNHN, MZUSP and USNPC.

########## Reference.

Fehlauer and Boeger (2005).

######## *Hamatopeduncularia* Yamaguti, 1953

######### Hamatopeduncularia cangatae

Taxon classificationAnimaliaDactylogyrideaDactylogyridae

Domingues, Soares & Watanabe, 2016

########## Type host.

*Aspistor quadriscutis* (Valenciennes, 1840)

########## Infection site.

Secondary lamellae of the gills.

########## Type locality.

Brazil, Pará State, Bragança, Fish market.

########## Holotype.

CHIOC 38264.

########## Paratypes.

CHIOC 38265 a–b, 38266.

########## Remarks.

Other paratypes deposited in the INPA collection.

########## Reference.

Domingues et al. (2016).

######## *Jainus* Mizelle, Kritsky & Crane, 1968

######### Jainus iocensis

Taxon classificationAnimaliaDactylogyrideaDactylogyridae

Cohen, Kohn & Boeger, 2012

########## Type host.

*Salminus brasiliensis*

########## Infection site.

Gills.

########## Type locality.

Brazil, Paraná River below and above of the reservoir of Itaipu Hydroelectric Power Station, Guaíra (24°04'48"S, 54°15'21"W).

########## Holotype.

CHIOC 37647 a.

########## Paratypes.

CHIOC 37606, 37635, 37637 a–c, 37638, 37640 a–b, 37644, 37645, 37646, 37647 b, 37650, 37651, 37653, 37655, 37670, 37672 a–c, 37673 a–b, 37675, 37687 a–b, 37690, 37695 a–c, 37700, 37702, 37703, 37704, 37708, 37710 a–b, 37712, 37723.

########## Reference.

Cohen et al. (2012).

######### Jainus piava

Taxon classificationAnimaliaDactylogyrideaDactylogyridae

Karling, Bellay, Takemoto & Pavanelli, 2011

########## Type host.

*Schizodon borellii* (Boulenger, 1900) (Osteichthyes: Anostomidae).

########## Infection site.

Gill filaments.

########## Type locality.

Brazil, upper Paraná River floodplain (22°50'–22°70'S, 53°15'–53°40'W).

########## Holotype.

CHIOC 37235 a.

########## Paratypes.

CHIOC 37235 b–c, 37236.

########## Reference.

Karling et al. (2011a).

######## *Kritskyia* Kohn, 1990

######### Kritskyia annakohnae

Taxon classificationAnimaliaDactylogyrideaDactylogyridae

Boeger, Tanaka & Pavanelli, 2001

########## Type host.

*Serrasalmus marginatus* Valenciennes, 1836

########## Infection site.

Urinary bladder and ureters.

########## Type locality.

Brazil, Paraná State, upper Paraná River, Baía River near Porto Rico.

########## Holotype.

CHIOC 34243 a.

########## Paratypes.

CHIOC 34243 b–m, 34244, 34245 a–b.

########## Remarks.

Paratypes CHIOC 34244 and 34245 a–b collected from *Serrasalmus spilopleura* Kner, 1858. Other paratypes deposited in the collections of INPA, HWML, MNHN and USNPC.

########## Reference.

Boeger et al. (2001).

######### Kritskyia boegeri

Taxon classificationAnimaliaDactylogyrideaDactylogyridae

Takemoto, Lizama & Pavanelli, 2002

########## Type host.

*Prochilodus lineatus*

########## Infection site.

Urinary bladder.

########## Type locality.

Brazil, upper Paraná River floodplain.

########## Holotype.

CHIOC 34701.

########## Paratypes.

CHIOC 34599, 34700.

########## Reference.

Takemoto et al. (2002).

######### Kritskyia eirasi

Taxon classificationAnimaliaDactylogyrideaDactylogyridae

Guidelli, Takemoto & Pavanelli, 2003

########## Type host.

*Leporinus lacustris* Campos, 1945 (Osteichthyes: Anostomidae).

########## Infection site.

Urinary bladder and ureters.

########## Type locality.

Brazil, upper Paraná River floodplain (22°50'–22°70'S, 53°15'–53°40'W).

########## Holotype.

CHIOC 36401.

########## Paratypes.

CHIOC 36396, 36397, 36398 a–b, 36399, 36400.

########## Reference.

Guidelli et al. (2003).

######### * Kritskyia moraveci

Taxon classificationAnimaliaDactylogyrideaDactylogyridae

Kohn, 1990

########## Type host.

*Rhamdia quelen*

########## Infection site.

Urinary bladder and ureters.

########## Type locality.

Brazil, Rio Grande do Sul State, reservoir of the Hydroelectric Power of Passo Fundo, São Valentim (27°32'S, 52°44'W).

########## Holotype.

CHIOC 32443 c.

########## Paratypes.

CHIOC 32443 a–b, d–r, 32444 a–b.

########## Reference.

Kohn (1990).

######### Kritskyia salmini

Taxon classificationAnimaliaDactylogyrideaDactylogyridae

Cepeda, Ceccarelli & Luque, 2011

########## Type host.

*Salminus brasiliensis*

########## Infection site.

Urinary bladder.

########## Type locality.

Brazil, Mato Grosso State, Pantanal wetlands, Cuiabá River (17°50'48"S, 57°24'6"W)

########## Holotype.

CHIOC 37533 a.

########## Paratypes.

CHIOC 37533 b–j.

########## Remarks.

Other paratypes deposited in the INPA collection.

########## Reference.

Cepeda et al. (2011).

######## *Ligophorus* Euzet & Suriano, 1977

######### Ligophorus brasiliensis

Taxon classificationAnimaliaDactylogyrideaDactylogyridae

Abdallah, Azevedo & Luque, 2009

########## Type host.

*Mugil liza* Valenciennes, 1836 (Osteichthyes: Mugilidae).

########## Infection site.

Gill lamellae.

########## Type locality.

Brazil, Rio de Janeiro State, Guandu River (22°48'32"S, 43°37'35"W).

########## Holotype.

CHIOC 37180 a.

########## Paratypes.

CHIOC 37180 b–d.

########## Reference.

Abdallah et al. (2009).

######### Ligophorus guanduensis

Taxon classificationAnimaliaDactylogyrideaDactylogyridae

Abdallah, Azevedo & Luque, 2009

########## Type host.

*Mugil liza*

########## Infection site.

Gill lamellae.

########## Type locality.

Brazil, Rio de Janeiro State, Guandu River (22°48'32"S, 43°37'35"W).

########## Holotype.

CHIOC 37181 a.

########## Paratypes.

CHIOC 37181 b–d.

########## Reference.

Abdallah et al. (2009).

######### Ligophorus lizae

Taxon classificationAnimaliaDactylogyrideaDactylogyridae

Abdallah, Azevedo & Luque, 2009

########## Type host.

*Mugil liza*

########## Infection site.

Gill lamellae.

########## Type locality.

Brazil, Rio de Janeiro State, Guandu River (22°48'32"S, 43°37'35"W).

########## Holotype.

CHIOC 37178 a.

########## Paratype.

CHIOC 37178 b.

########## Reference.

Abdallah et al. (2009).

######### Ligophorus tainhae

Taxon classificationAnimaliaDactylogyrideaDactylogyridae

Abdallah, Azevedo & Luque, 2009

########## Type host.

*Mugil liza*

########## Infection site.

Gill lamellae.

########## Type locality.

Brazil, Rio de Janeiro State, Guandu River (22°48'32"S, 43°37'35"W).

########## Holotype.

CHIOC 37179 a.

########## Paratypes.

CHIOC 37179 b–d.

########## Reference.

Abdallah et al. (2009).

######## *Marumbius* Boeger, Ferreira, Vianna & Patella, 2014

######### Marumbius amplexus

Taxon classificationAnimaliaDactylogyrideaDactylogyridae

Boeger, Ferreira, Vianna & Patella, 2014

########## Type host.

*Characidium lanei*

########## Infection site.

Gills.

########## Type locality.

Brazil, Paraná State, Morretes, Marumbi River (25°29'27"S, 45°49'67"W).

########## Holotype.

CHIOC 37897.

########## Paratype.

CHIOC 37896.

########## Remarks.

Other paratypes deposited in the collections of IPCAS and USNPC.

########## Reference.

Boeger et al. (2014).

######### * Marumbius dorsivaginatus

Taxon classificationAnimaliaDactylogyrideaDactylogyridae

Boeger, Ferreira, Vianna & Patella, 2014

########## Type host.

*Characidium pterostictum* Gomes, 1947

########## Infection site.

Gills.

########## Type locality.

Brazil, Paraná State, Morretes, Marumbi River (25°29'27"S, 45°49'67"W).

########## Holotype.

CHIOC 37893.

########## Paratypes.

CHIOC 37884, 37885, 37886, 37887, 37888, 37889, 37890, 37891, 37892, 37894, 37895.

########## Remarks.

Other paratypes deposited in the collections of IPCAS, HWML and USNPC.

########## Reference.

Boeger et al. (2014).

######## *Mexicana* Caballero & Bravo-Hollis, 1959

######### Mexicana anisotremum

Taxon classificationAnimaliaDactylogyrideaDactylogyridae

Cezar, Paschoal & Luque, 2012

########## Type host.

*Anisotremus virginicus* (Linnaeus, 1758) (Osteichthyes: Haemulidae).

########## Infection site.

Gills.

########## Type locality.

Brazil, coastal zone of Rio de Janeiro State (21–23°S, 42–45°W).

########## Holotype.

CHIOC 37767.

########## Paratypes.

CHIOC 37768, 37769.

########## Remarks.

Paratype CHIOC 37769 collected from *Anisotremus surinamensis* (Bloch, 1791).

########## Reference.

Cezar et al. (2012).

######### Mexicana atlantica

Taxon classificationAnimaliaDactylogyrideaDactylogyridae

Luque, Amato & Takemoto, 1992

########## Type host.

*Haemulon steindachneri* (Jordan & Gilbert, 1882) (Osteichthyes: Haemulidae).

########## Infection site.

Distal portion of gill filaments.

########## Type locality.

Brazil, Rio de Janeiro State, Sepetiba Bay, Itacuruçá (22°51'S, 43°56'W).

########## Paratypes.

CHIOC 33109, 33110.

########## Remarks.

Holotype and other paratypes deposited in the USNM collection.

########## Reference.

Luque et al. (1992).

######## *Mymarothecium* Kritsky, Boeger & Jégu, 1996

######### Mymarothecium boegeri

Taxon classificationAnimaliaDactylogyrideaDactylogyridae

Cohen & Kohn, 2005

########## Type host.

*Colossoma macropomum* (Cuvier, 1816) (Osteichthyes: Serrasalmidae).

########## Infection site.

Gills.

########## Type locality.

Brazil, Ceará State, Pentecoste, Aquaria of the Research Center in Aquiculture “Rodolfo von Ihering, DNOCS”.

########## Holotype.

CHIOC 36453.

########## Paratypes.

CHIOC 36454, 36455, 36456, 36457.

########## Reference.

Cohen and Kohn (2005).

######### Mymarothecium ianwhittingtoni

Taxon classificationAnimaliaDactylogyrideaDactylogyridae

Leão, São Clemente & Cohen, 2015

########## Type host.

*Piaractus mesopotamicus*

########## Infection site.

Gills.

########## Type locality.

Brazil, Paraná State, Paraná River, Toledo (24°44'29.0"S, 53°44'51.2"W).

########## Holotype.

CHIOC 37906 a.

########## Paratypes.

CHIOC 37906 b–d.

########## Remarks.

Other paratypes deposited in the INPA collection.

########## Reference.

Leão et al. (2015).

######### Mymarothecium viatorum

Taxon classificationAnimaliaDactylogyrideaDactylogyridae

Boeger, Piasecki & Sobecka, 2002

########## Type host.

*Piaractus brachypomus* (Cuvier, 1818)

########## Infection site.

Gills.

########## Type locality.

Poland, Szczecin, Odra River.

########## Holotype.

CHIOC 35041.

########## Paratypes.

CHIOC 35042 a–i.

########## Remarks.

Other paratypes deposited in the collections of INPA, HWML and USNPC.

########## Reference.

Boeger et al. (2002).

######## *Notozothecium* Boeger & Kritsky, 1988

######### Notozothecium lamotheargumedoi

Taxon classificationAnimaliaDactylogyrideaDactylogyridae

Cohen & Kohn, 2008

########## Type host.

*Rhaphiodon vulpinus* Spix & Agassiz, 1829 (Osteichthyes: Cynodontidae).

########## Infection site.

Gills.

########## Type locality.

Brazil, Paraná State, inside and outside of the reservoir of Itaipu Hydroelectric Power Station, Foz do Iguaçú (25°32'52"S, 54°35'17"W).

########## Holotype.

CHIOC 36895.

########## Paratypes.

CHIOC 36896 a–b, 36897 a–d, 36898, 36899 a–d, 36900 a–s, 36901 a–e.

########## Remarks.

Paratypes collected in different localities of the reservoir of Itaipu Hydroelectric Power Station: CHIOC 36897 a–d and 36898 (Santa Helena, 24°51'37"S, 54°19'58"W), and 36899 a–d, 36900 a–d and 36901 a–e (Guaíra, 24°04'48"S, 54°15'21"W).

########## Reference.

Cohen and Kohn (2008b).

######## *Parancylodiscoides* Caballero & Bravo-Hollis, 1960

######### Parancylodiscoides caballerobravorum

Taxon classificationAnimaliaDactylogyrideaDactylogyridae

Cezar, Luque & Amato, 1999

########## Type host.

*Chaetodipterus faber*

########## Infection site.

Gill filaments.

########## Type locality.

Brazil, coastal zone of Rio de Janeiro State (21–23°S, 41–45°W).

########## Holotype.

CHIOC 34001 a.

########## Paratypes.

CHIOC 34001 b–d.

########## Remarks.

Collection numbers were not cited in the original description. Other paratypes deposited in CNHE. Kritsky (2012) considered the species to be a synonym of *Parancylodiscoides longiphallus* (MacCallum, 1915).

########## References.

Cezar et al. (1999), Kritsky (2012).

######## *Pavanelliella* Kritsky & Boeger, 1998

######### Pavanelliella laertei

Taxon classificationAnimaliaDactylogyrideaDactylogyridae

Aguiar, Ceccarelli & Luque, 2011

########## Type host.

*Pimelodus microstoma* Steindachner, 1877 (= *Pimelodus heraldoi* Azpelicueta, 2001) (Osteichthyes: Pimelodidae).

########## Infection site.

Nasal cavity.

########## Type locality.

Brazil, São Paulo State, Pirassununga, Cachoeira de Emas, Mogi Guaçu River (21°58'S, 47°26'W).

########## Holotype.

CHIOC 37561.

########## Paratypes.

CHIOC 37562, 37563, 37564, 37565, 37566.

########## Reference.

Aguiar et al. (2011).

######### Pavanelliella takemotoi

Taxon classificationAnimaliaDactylogyrideaDactylogyridae

Aguiar, Ceccarelli & Luque, 2011

########## Type host.

*Pimelodus maculatus* Lacepède, 1803

########## Infection site.

Nasal cavity.

########## Type locality.

Brazil, São Paulo State, Pirassununga, Cachoeira de Emas, Mogi Guaçu River (21°58'S, 47°26'W).

########## Holotype.

CHIOC 37567 a.

########## Paratypes.

CHIOC 37567 b, 37568 a–f, 37569, 37570 a–b, 37571, 37572, 37573, 37574, 37575 a–b, 37576 a–b, 37577 a–b, 37578, 37579 a–b, 37580, 37582 a–c.

########## Reference.

Aguiar et al. (2011).

######## *Philocorydoras* Suriano, 1986

######### Philocorydoras longus

Taxon classificationAnimaliaDactylogyrideaDactylogyridae

Yamada, Brandão, Yamada & Silva, 2015

########## Type host.

*Hoplosternum littorale* (Hancock, 1828) (Osteichthyes: Callichthyidae).

########## Infection site.

Gills.

########## Type locality.

Brazil, São Paulo State, Taquarituba, upper Paranapanema River, Jurumirim Reservoir (23°12'17"S, 49°13'19"W).

########## Holotype.

CHIOC 38205.

########## Paratype.

CHIOC 38206.

########## Reference.

Yamada et al. (2015).

######## *Protogyrodactylus* Johnston & Tiegs, 1922

######### Protogyrodactylus ethiopicus

Taxon classificationAnimaliaDactylogyrideaDactylogyridae

Boeger, Diamaka, Pariselle & Patella, 2012

########## Type host.

*Gerres nigri* Günther, 1859 (Osteichthyes: Gerreidae).

########## Infection site.

Gills.

########## Type locality.

Senegal, Bamboung, Sine–Saloum River (13°49'30.5"N, 16°31'44"W).

########## Paratypes.

CHIOC 37744 a–n.

########## Remarks.

Holotype deposited in the MNHN collection. Other paratypes deposited in MNHN and USNPC.

########## Reference.

Boeger et al. (2012).

######### Protogyrodactylus kritskyi

Taxon classificationAnimaliaDactylogyrideaDactylogyridae

Boeger, Diamaka, Pariselle & Patella, 2012

########## Type host.

*Gerres nigri*

########## Infection site.

Gills.

########## Type locality.

Senegal, Bamboung, Sine–Saloum River (13°49'30.5"N, 16°31'44"W).

########## Paratypes.

CHIOC 37743 a–k.

########## Remarks.

Holotype deposited in the MNHN collection. Other paratypes deposited in MNHN and USNPC.

########## Reference.

Boeger et al. (2012).

######## *Protorhinoxenus* Domingues & Boeger, 2002

######### * Protorhinoxenus prochilodi

Taxon classificationAnimaliaDactylogyrideaDactylogyridae

Domingues & Boeger, 2002

########## Type host.

*Prochilodus lineatus*

########## Infection site.

Gill filaments.

########## Type locality.

Brazil, Paraná State, metropolitan area of Curitiba, Campina Grande do Sul, reservoir of Capivari–Cachoeira.

########## Holotype.

CHIOC 34542.

########## Paratype.

CHIOC 34543.

########## Remarks.

Other paratypes deposited in the collections of INPA and IPCR.

########## Reference.

Domingues and Boeger (2002).

######## *Pseudempleurosoma* Yamaguti, 1965

######### Pseudempleurosoma gibsoni

Taxon classificationAnimaliaDactylogyrideaDactylogyridae

Santos, Mourão & Cárdenas, 2001

########## Type host.

*Paralonchurus brasiliensis* (Steindachner, 1875) (Osteichthyes: Sciaenidae).

########## Infection site.

Oesophagous.

########## Type locality.

Brazil, São Paulo State, off Ubatuba (23°35'S, 045°06'W).

########## Holotype.

CHIOC 34337 a.

########## Paratypes.

CHIOC 34337 b–e, 34338 a–b.

########## Reference.

Santos et al. (2001).

######### Pseudempleurosoma guanabarensis

Taxon classificationAnimaliaDactylogyrideaDactylogyridae

Carvalho & Luque, 2012

########## Type host.

*Trichiurus lepturus*

########## Infection site.

Oesophagous.

########## Type locality.

Brazil, Rio de Janeiro State, Guanabara Bay (23°1'52"S, 43°11'56"W).

########## Holotype.

CHIOC 37389.

########## Paratypes.

CHIOC 37390 a–c, 37391.

########## Reference.

Carvalho and Luque (2012).

######## *Rhinonastes* Kritsky, Thatcher & Boeger, 1988

######### Rhinonastes curimatae

Taxon classificationAnimaliaDactylogyrideaDactylogyridae

Monteiro & Brasil-Sato, 2014

########## Type host.

*Prochilodus argenteus*

########## Infection site.

Nasal cavity.

########## Type locality.

Brazil, Minas Gerais State, São Francisco River near Três Marias (18°12'32"S, 45°14'41"W).

########## Holotype.

CHIOC 37953 a.

########## Paratypes.

CHIOC 37953 b–c, 37954 a–f, 37955.

########## Reference.

Monteiro and Brasil-Sato (2014b).

######## *Rhinoxenus* Kritsky, Thatcher & Boeger, 1988

######### Rhinoxenus anaclaudiae

Taxon classificationAnimaliaDactylogyrideaDactylogyridae

Domingues & Boeger, 2005

########## Type host.

Triportheus cf. nematurus (Kner, 1858)

########## Infection site.

Nasal cavity.

########## Type locality.

Brazil, Mato Grosso do Sul State, Miranda River, Passo do Lontra.

########## Holotype.

CHIOC 36300.

########## Paratypes.

CHIOC 36301 a–e.

########## Remarks.

Other paratypes deposited in INPA, MZUSP, MNHN and USNPC.

########## Reference.

Domingues and Boeger (2005).

######### Rhinoxenus bulbovaginatus

Taxon classificationAnimaliaDactylogyrideaDactylogyridae

Boeger, Domingues & Pavanelli, 1995

########## Type host.

*Salminus brasiliensis*

########## Infection site.

Nasal cavities.

########## Type locality.

Brazil, Paraná State, Paraná River basin near Porto Rico.

########## Holotype.

CHIOC 33269 a.

########## Paratypes.

CHIOC 33269 b–e.

########## Remarks.

Other paratypes deposited in the collections of HWML and USNM.

########## Reference.

Boeger et al. (1995a).

######### Rhinoxenus curimbatae

Taxon classificationAnimaliaDactylogyrideaDactylogyridae

Domingues & Boeger, 2005

########## Type host.

Prochilodus cf. lineatus

########## Infection site.

Nasal cavity.

########## Type locality.

Brazil, Paraná State, metropolitan area of Curitiba, Campina Grande do Sul, reservoir of Capivari–Cachoeira.

########## Holotype.

CHIOC 36303.

########## Paratypes.

CHIOC 36304 a–b.

########## Remarks.

Other paratypes deposited in the collections of INPA and MNHN.

########## Reference.

Domingues and Boeger (2005).

######### Rhinoxenus euryxenus

Taxon classificationAnimaliaDactylogyrideaDactylogyridae

Domingues & Boeger, 2005

########## Type host.

*Serrasalmus marginatus*

########## Infection site.

Nasal cavity.

########## Type locality.

Brazil, Mato Grosso do Sul State, Paraná River, Medalha Bay.

########## Holotype.

CHIOC 36293.

########## Paratypes.

CHIOC 36294 a–g.

########## Remarks.

Other paratypes deposited in the collections of INPA and MNHN.

########## Reference.

Domingues and Boeger (2005).

######### Rhinoxenus guianensis

Taxon classificationAnimaliaDactylogyrideaDactylogyridae

Domingues & Boeger, 2005

########## Type host.

*Curimata cyprinoides* (Linnaeus, 1766) (Osteichthyes: Curimatidae).

########## Infection site.

Nasal cavity.

########## Type locality.

French Guiana, Degrad Forian, Iracoubo.

########## Holotype.

CHIOC 36305.

########## Paratypes.

CHIOC 36306 a–d.

########## Remarks.

Other paratypes deposited in the collections of INPA, MNHN and USNPC.

########## Reference.

Domingues and Boeger (2005).

######## *Sciadicleithrum* Kritsky, Thatcher & Boeger, 1989

######### Sciadicleithrum araguariensis

Taxon classificationAnimaliaDactylogyrideaDactylogyridae

Paschoal, Scholz, Tavares-Dias & Luque, 2016

########## Type host.

*Crenicichla labrina* (Spix & Agassiz, 1831) (Osteichthyes: Cichlidae).

########## Infection site.

Gills.

########## Type locality.

Brazil, Amapá State, Ferreira Gomes, Araguari River (0°52'N, 51°12'W).

########## Holotype.

CHIOC 38091 a.

########## Paratypes.

CHIOC 38091 b–d.

########## Remarks.

Other paratype deposited in the IPCA collection.

########## Reference.

Paschoal et al. (2016).

######### Sciadicleithrum edgari

Taxon classificationAnimaliaDactylogyrideaDactylogyridae

Paschoal, Scholz, Tavares-Dias & Luque, 2016

########## Type host.

*Satanoperca jurupari* (Heckel, 1840) (Osteichthyes: Cichlidae).

########## Infection site.

Gills.

########## Type locality.

Brazil, Amapá State, Ferreira Gomes, Araguari River (0°52'N, 51°12'W).

########## Holotype.

CHIOC 38092 a.

########## Paratypes.

CHIOC 38092 b–c.

########## Remarks.

Other paratype deposited in the IPCA collection.

########## Reference.

Paschoal et al. (2016).

######### Sciadicleithrum frequens

Taxon classificationAnimaliaDactylogyrideaDactylogyridae

Bellay, Takemoto, Yamada & Pavanelli, 2008

########## Type host.

*Geophagus brasiliensis* (Quoy & Gaimard, 1824) (Osteichthyes: Cichlidae).

########## Infection site.

Gills.

########## Type locality.

Brazil, Paraná State, Ivaí River basin, Mourão Reservoir (24°02'S, 52°22'W).

########## Holotype.

CHIOC 36924.

########## Paratypes.

CHIOC 36921 a–c, 36922, 36923.

########## Remarks.

Paratypes collected in other reservoirs: CHIOC 36921 a–c (Iguaçu River basin, Iraí, 25°25'S, 49°06'W) and 36922 (Piriqui River basin, Melissa, 24°48'S, 53°18'W). Other paratype deposited in the BMNH collection.

########## Reference.

Bellay et al. (2008).

######### Sciadicleithrum guanduensis

Taxon classificationAnimaliaDactylogyrideaDactylogyridae

Carvalho, Tavares & Luque, 2008

########## Type host.

*Geophagus brasiliensis*

########## Infection site.

Gills.

########## Type locality.

Brazil, Rio de Janeiro State, Seropédica, Guandu River (22°48'32"S, 43°37'35"W).

########## Holotype.

CHIOC 36989 a.

########## Paratypes.

CHIOC 36989 b–f.

########## Reference.

Carvalho et al. (2008).

######### Sciadicleithrum joanae

Taxon classificationAnimaliaDactylogyrideaDactylogyridae

Yamada, Takemoto, Bellay & Pavanelli, 2009

########## Type host.

*Crenicichla niederleinii* Holmberg, 1891

########## Infection site.

Gill filaments.

########## Type locality.

Brazil, Paraná, Paraná River (22°50'–22°70'S, 53°15'–53°40'W).

########## Holotype.

CHIOC 37161.

########## Paratypes.

CHIOC 37162, 37163 a–b, 37164.

########## Reference.

Yamada et al. (2009).

######### Sciadicleithrum jurupari

Taxon classificationAnimaliaDactylogyrideaDactylogyridae

Melo, Santos & Santos, 2012

########## Type host.

*Satanoperca jurupari*

########## Infection site.

Gills.

########## Type locality.

Brazil, Pará State, Belém, Guamá River (01°27'21"S, 48°30'14"W).

########## Holotype.

CHIOC 37766 a.

########## Paratypes.

CHIOC 37766 b–f.

########## Reference.

Melo et al. (2012).

######### Sciadicleithrum kritskyi

Taxon classificationAnimaliaDactylogyrideaDactylogyridae

Bellay, Takemoto, Yamada & Pavanelli, 2009

########## Type host.

*Geophagus proximus* (Castelnau, 1855)

########## Infection site.

Gill filaments.

########## Type locality.

Brazil, Paraná State, upper Paraná River floodplain, Porto Rico (22°50'–22°70'S, 53°15'–53°40'W).

########## Holotype.

CHIOC 37188.

########## Paratypes.

CHIOC 37189, 37190, 37191.

########## Reference.

Bellay et al. (2009).

######### Sciadicleithrum paranaensis

Taxon classificationAnimaliaDactylogyrideaDactylogyridae

Bellay, Takemoto, Yamada & Pavanelli, 2009

########## Type host.

*Geophagus proximus*

########## Infection site.

Gill filaments.

########## Type locality.

Brazil, Paraná State, upper Paraná River floodplain, Porto Rico (22°50'–22°70'S, 53°15'–53°40'W).

########## Holotype.

CHIOC 37183.

########## Paratypes.

CHIOC 37184, 37185, 37186, 37187.

########## Reference.

Bellay et al. (2009).

######### Sciadicleithrum satanopercae

Taxon classificationAnimaliaDactylogyrideaDactylogyridae

Yamada, Takemoto, Bellay & Pavanelli, 2009

########## Type host.

*Satanoperca pappaterra* (Heckel, 1840)

########## Infection site.

Gill filaments.

########## Type locality.

Brazil, Paraná State, upper Paraná River floodplain (22°50'–22°70'S, 53°15'–53°40'W).

########## Holotype.

CHIOC 37165 a.

########## Paratypes.

CHIOC 37165 b, 37166 a–b, 37167.

########## Reference.

Yamada et al. (2009).

######## *Susanlimae* Boeger, Pariselle & Patella, 2015

######### * Susanlimae ianwhittingtoni

Taxon classificationAnimaliaDactylogyrideaDactylogyridae

Boeger, Pariselle & Patella, 2015

########## Type host.

*Pseudeutropius moolenburghae* Weber & de Beaufort, 1913 (Osteichthyes: Schilbeidae).

########## Infection site.

Gill rakers.

########## Type locality.

Indonesia, Jambi Province, Pemayung Subdistrict, Batang Hari River, near Kubu Kandang village (1°36'17.51"S, 103°19'16.65"E).

########## Holotype.

CHIOC 38202 a.

########## Paratypes.

CHIOC 38202 b–c.

########## Remarks.

Two paratypes deposited in MNHN and one paratype kept in the private collection of Antoine Pariselle.

########## Reference.

Boeger et al. (2015).

######## *Telethecium* Kritsky, Van Every & Boeger, 1996

######### * Telethecium nasalis

Taxon classificationAnimaliaDactylogyrideaDactylogyridae

Kritsky, Van Every & Boeger, 1996

########## Type host.

*Osteoglossum bicirrhosum* (Vandelli, 1829) (Osteichthyes: Osteoglossidae).

########## Infection site.

Nasal cavity.

########## Type locality.

Brazil, Amazonas State, Furo do Catalão near Manaus.

########## Holotype.

CHIOC 33639.

########## Remarks.

Paratypes deposited in HWML and USNPC.

########## Reference.

Kritsky et al. (1996).

######### Telethecium paniculum

Taxon classificationAnimaliaDactylogyrideaDactylogyridae

Kritsky, Van Every & Boeger, 1996

########## Type host.

*Pellona flavipinnis* (Valenciennes, 1837) (Osteichthyes: Pristigasteridae).

########## Infection site.

Nasal cavity.

########## Type locality.

Brazil, Amazonas State, Solimões River, Marchantaria Island near Manaus.

########## Holotype.

CHIOC 33640.

########## Remarks.

Paratypes deposited in HWML and USNPC.

########## Reference.

Kritsky et al. (1996).

######## *Tereancistrum* Kritsky, Thatcher & Kayton, 1980

######### Tereancistrum arcuatus

Taxon classificationAnimaliaDactylogyrideaDactylogyridae

Cohen, Kohn & Boeger, 2012

########## Type host.

*Salminus brasiliensis*

########## Infection site.

Gills.

########## Type locality.

Brazil, Paraná State, Paraná River below and above of the reservoir of Itaipu Hydroelectric Power Station, Guaíra (24°04'48"S, 54°15'21"W).

########## Holotype.

CHIOC 37628.

########## Paratypes.

CHIOC 37609, 37665, 37674 a–b, 37678.

########## Reference.

Cohen et al. (2012).

######### Tereancistrum curimba

Taxon classificationAnimaliaDactylogyrideaDactylogyridae

Lizama, Takemoto & Pavanelli, 2004

########## Type host.

*Prochilodus lineatus*

########## Infection site.

Gills.

########## Type locality.

Brazil, upper Paraná River floodplain (22°43'S, 53°10'W).

########## Holotype.

CHIOC 36224.

########## Paratypes.

CHIOC 36225, 36226, 36227 a–b, 36228.

########## Remarks.

Other paratypes deposited in BMNH and USNPC.

########## Reference.

Lizama et al. (2004).

######### Tereancistrum paranaensis

Taxon classificationAnimaliaDactylogyrideaDactylogyridae

Karling, Lopes, Takemoto & Pavanelli, 2014

########## Type host.

*Schizodon borellii*

########## Infection site.

Gill filaments.

########## Type locality.

Brazil, upper Paraná River floodplain (22°50'–22°70'S, 53°15'–53°40'W).

########## Holotype.

CHIOC 37868.

########## Paratypes.

CHIOC 37866, 37867.

########## Reference.

Karling et al. (2014).

######### Tereancistrum pirassununguensis

Taxon classificationAnimaliaDactylogyrideaDactylogyridae

Cepeda, Ceccarelli & Luque, 2012

########## Type host.

*Prochilodus lineatus*

########## Infection site.

Gills.

########## Type locality.

Brazil, São Paulo State, Pirassununga, Mogi Guaçu River (21°55'32.49"S, 47°22'13.76"W).

########## Holotype.

CHIOC 37816 a.

########## Paratypes.

CHIOC 37816 b–j.

########## Reference.

Cepeda et al. (2012).

######### Tereancistrum toksonum

Taxon classificationAnimaliaDactylogyrideaDactylogyridae

Lizama, Takemoto & Pavanelli, 2004

########## Type host.

*Prochilodus lineatus*

########## Infection site.

Gills.

########## Type locality.

Brazil, upper Paraná River floodplain (22°43'S, 53°10'W).

########## Holotype.

CHIOC 36229.

########## Paratypes.

CHIOC 36230 a–b, 36231.

########## Remarks.

Other paratypes deposited in BMNH and USNPC.

########## Reference.

Lizama et al. (2004).

######## *Trinibaculum* Kritsky, Thatcher & Kayton, 1980

######### Trinibaculum rotundus

Taxon classificationAnimaliaDactylogyrideaDactylogyridae

Karling, Lopes, Takemoto & Pavanelli, 2011

########## Type host.

*Schizodon borellii*

########## Infection site.

Gill filaments.

########## Type locality.

Brazil, upper Paraná River floodplain (22°50'–22°70'S, 53°15'–53°40'W).

########## Holotype.

CHIOC 37529 a.

########## Paratypes.

CHIOC 37529 b–e.

########## Reference.

Karling et al. (2011b).

######## *Trinigyrus* Hanek, Molnar & Fernando, 1974

######### Trinigyrus mourei

Taxon classificationAnimaliaDactylogyrideaDactylogyridae

Boeger & Belmont-Jégu, 1994

########## Type host.

*Squaliforma emarginata* (Valenciennes, 1840) [= *Hypostomus emarginatus*] (Osteichthyes: Loricariidae).

########## Infestation site.

Body surface.

########## Type locality.

Brazil, Amazonas State, Manaus, Bairro São Jorge.

########## Holotype.

CHIOC 33052 a.

########## Paratypes.

CHIOC 33052 b–d.

########## Remarks.

Other paratypes deposited in the collections of HWML and USNM.

########## Reference.

Boeger and Belmont-Jégu (1994).

######## *Unilatus* Mizelle & Kritsky, 1967

######### Unilatus irae

Taxon classificationAnimaliaDactylogyrideaDactylogyridae

Branches & Domingues, 2014

########## Type host.

*Leporacanthicus galaxias* Isbrücker & Nijssen, 1989 (Osteichthyes: Loricariidae).

########## Infection site.

Gills.

########## Type locality.

Brazil, Pará State, Guamá River, Capitão Poço (1°34.465'S, 47°02.063'W).

########## Holotype.

CHIOC 37861 a.

########## Paratypes.

CHIOC 37861 b–k.

########## Remarks.

Other paratypes deposited in INPA, MPEG and USNPC.

########## Reference.

Branches and Domingues (2014).

######## *Urocleidoides* Mizelle & Price, 1964

######### Urocleidoides aimarai

Taxon classificationAnimaliaDactylogyrideaDactylogyridae

Moreira, Scholz & Luque, 2015

########## Type host.

*Hoplias aimara* (Valenciennes, 1847) (Osteichthyes: Erythrinidae).

########## Infection site.

Gills.

########## Type locality.

Brazil, Pará State, Xingu River around Altamira (3°12'S, 52°12'W).

########## Holotype.

CHIOC 38001 a.

########## Paratypes.

CHIOC 38001 b–d.

########## Remarks.

Two paratypes deposited in the IPCAS collection.

########## Reference.

Moreira et al. (2015).

######### Urocleidoides brasiliensis

Taxon classificationAnimaliaDactylogyrideaDactylogyridae

Rosim, Mendoza-Franco & Luque, 2011

########## Type host.

*Hoplias malabaricus* (Bloch, 1794)

########## Infection site.

Gill lamellae.

########## Type locality.

Brazil, Mato Grosso State, Cuiabá (16°58'N, 56°25'W).

########## Holotype.

CHIOC 37470 a.

########## Paratypes.

CHIOC 37470 b–f.

########## Reference.

Rosim et al. (2011).

######### Urocleidoides cuiabai

Taxon classificationAnimaliaDactylogyrideaDactylogyridae

Rosim, Mendoza-Franco & Luque, 2011

########## Type host.

*Hoplias malabaricus*

########## Infection site.

Gill lamellae.

########## Type locality.

Brazil, Mato Grosso State, Cuiabá (16°58'N, 56°25'W).

########## Holotype.

CHIOC 37469 a.

########## Paratypes.

CHIOC 37469 b–e.

########## Remarks.

Other paratypes deposited in CNHE.

########## Reference.

Rosim et al. (2011).

######### Urocleidoides malabaricusi

Taxon classificationAnimaliaDactylogyrideaDactylogyridae

Rosim, Mendoza-Franco & Luque, 2011

########## Type host.

*Hoplias malabaricus*

########## Infection site.

Gill lamellae.

########## Type locality.

Brazil, Mato Grosso State, Cuiabá (16°58'N, 56°25'W).

########## Holotype.

CHIOC 37466 a.

########## Paratypes.

CHIOC 37466 b–c.

########## Reference.

Rosim et al. (2011).

######### Urocleidoides naris

Taxon classificationAnimaliaDactylogyrideaDactylogyridae

Rosim, Mendoza-Franco & Luque, 2011

########## Type host.

*Hoplias malabaricus*

########## Infection site.

Nasal cavities.

########## Type locality.

Brazil, Mato Grosso State, Cuiabá (16°58'N, 56°25'W).

########## Holotype.

CHIOC 37468 a.

########## Paratypes.

CHIOC 37468 b–k.

########## Remarks.

Other paratypes deposited in CNHE.

########## Reference.

Rosim et al. (2011).

######### Urocleidoides xinguensis

Taxon classificationAnimaliaDactylogyrideaDactylogyridae

Moreira, Scholz & Luque, 2015

########## Type host.

*Hoplias aimara*

########## Infection site.

Gills.

########## Type locality.

Brazil, Pará State, Xingu River around Altamira (3°12'S, 52°12'W).

########## Holotype.

CHIOC 38002 a.

########## Paratypes.

CHIOC 38002 b–d.

########## Remarks.

Two paratypes deposited in the IPCAS collection.

########## Reference.

Moreira et al. (2015).

######## *Whittingnocotyle* Santos Neto, Rodrigues & Domingues, 2015

######### * Whittingnocotyle caetei

Taxon classificationAnimaliaDactylogyrideaDactylogyridae

Santos Neto, Rodrigues & Domingues, 2015

########## Type host.

*Hoplerythrinus unitaeniatus* (Agassiz, 1829) (Osteichthyes: Erythrinidae).

########## Infection site.

Gills.

########## Type locality.

Brazil, Pará State, Augusto Corrêa, Caeté River (01°3'58.21"S, 46°40'3.65"W).

########## Holotype.

CHIOC 38012 a.

########## Paratypes.

CHIOC 38012 b–d.

########## Remarks.

Other paratypes deposited in INPA and MPEG.

########## Reference.

Santos Neto et al. (2015).

######### Whittingnocotyle jeju

Taxon classificationAnimaliaDactylogyrideaDactylogyridae

Santos Neto, Rodrigues & Domingues, 2015

########## Type host.

*Hoplerythrinus unitaeniatus*

########## Infection site.

Gills.

########## Type locality.

Brazil, Pará State, Irituia, Guamá River (01°51'59.8"S, 47°24'17.2"W).

########## Holotype.

CHIOC 38014 a.

########## Paratypes.

CHIOC 38014 b–e.

########## Remarks.

Other paratypes deposited in INPA and MPEG.

########## Reference.

Santos Neto et al. (2015).

####### Diplectanidae Monticelli, 1903

######## *Acleotrema* Johnston & Tiegs, 1922

######### Acleotrema lamothei

Taxon classificationAnimaliaDactylogyrideaDiplectanidae

Santos, Bianchi & Gibson, 2008

########## Type host.

*Kyphosus incisor* (Cuvier, 1831) (Osteichthyes: Kyphosidae).

########## Infection site.

Gills.

########## Type locality.

Brazil, off Rio de Janeiro State, Ilha Grande Bay (23°00'–23°40'S, 44°00'–44°40'W)

########## Holotype.

CHIOC 36926 a.

########## Paratypes.

CHIOC 36926 b–d.

########## Reference.

Santos et al. (2008).

######## *Anoplectanum* Boeger, Fehlauer & Marques, 2006

######### * Anoplectanum haptorodynatum

Taxon classificationAnimaliaDactylogyrideaDiplectanidae

Boeger, Fehlauer & Marques, 2006

########## Type host.

*Petilipinnis grunniens* (Jardine & Schomburgk, 1843) (Osteichthyes: Sciaenidae).

########## Infection site.

Gills.

########## Type locality.

Brazil, Tocantins State, Tocantins River, Porto Nacional (10°41'45"S, 48°25'54"W).

########## Paratypes.

CHIOC 36504 a–b.

########## Remarks.

Paratypes from CHIOC collected in the Tocantins River, Santa Helena (Tocantins State). Holotype deposited in MZUSP. Other paratypes deposited in INPA, MZUSP, MNHN and USNPC.

########## Reference.

Boeger et al. (2006).

######### Anoplectanum microsoma

Taxon classificationAnimaliaDactylogyrideaDiplectanidae

Boeger, Fehlauer & Marques, 2006

########## Type host.

*Pachyurus junki*

########## Infection site.

Gills.

########## Type locality.

Brazil, Tocantins State, Tocantins River, Santa Helena.

########## Paratypes.

CHIOC 36503 a–b.

########## Remarks.

Holotype deposited in MZUSP. Other paratypes deposited in INPA, MZUSP, MNHN and USNPC.

########## Reference.

Boeger et al. (2006).

######## *Darwinoplectanum* Domingues, Diamanka & Pariselle, 2011

######### Darwinoplectanum amphiatlanticus

Taxon classificationAnimaliaDactylogyrideaDiplectanidae

Domingues, Diamanka & Pariselle, 2011

########## Type host.

*Eucinostomus melanopterus* (Bleecker, 1863) (Osteichthyes: Gerreidae).

########## Infection site.

Gills.

########## Type locality.

Senegal, Sine Saloum, Bamboung (13°49'30.5"N, 16°31'44"W).

########## Holotype.

CHIOC 37546 a.

########## Paratypes.

CHIOC 37546 b–d.

########## Remarks.

Other paratypes deposited in INPA, MPEG and USNPC.

########## Reference.

Domingues et al. (2011).

######### * Darwinoplectanum figueiredoi

Taxon classificationAnimaliaDactylogyrideaDiplectanidae

Domingues, Diamanka & Pariselle, 2011

########## Type host.

*Eucinostomus argenteus* Baird & Girard, 1855

########## Infection site.

Gills.

########## Type locality.

Brazil, Paraná State, Pontal do Paraná (25°35'28.27"S, 48°21'10.70"W).

########## Holotype.

CHIOC 37545 a.

########## Paratypes.

CHIOC 37545 b–f.

########## Remarks.

Other paratypes deposited in INPA, MPEG and USNPC.

########## Reference.

Domingues et al. (2011).

######## *Diplectanum* Diesing 1858

######### Diplectanum copiosum

Taxon classificationAnimaliaDactylogyrideaDiplectanidae

Boeger, Fehlauer & Marques, 2006

########## Type host.

*Pachyurus junki*

########## Infection site.

Gills.

########## Type locality.

Brazil, Tocantins State, Tocantins River, Santa Helena.

########## Paratypes.

CHIOC 36501 a–m.

########## Remarks.

Holotype deposited in MZUSP. Other paratypes deposited in INPA, MZUSP and USNPC.

########## Reference.

Boeger et al. (2006).

######### Diplectanum monticelli

Taxon classificationAnimaliaDactylogyrideaDiplectanidae

Domingues & Boeger, 2003

########## Type host.

*Cynoscion leairchus* (Cuvier, 1830) (Osteichthyes: Sciaenidae).

########## Infection site.

Gills.

########## Type locality.

Brazil, Rio de Janeiro State, Itacuruçá (22°55'S, 43°55'W).

########## Holotype.

CHIOC 34962.

########## Paratypes.

CHIOC 34963 a–b, 34964 a–c.

########## Remarks.

Other paratypes deposited in HWML and USNPC.

########## Reference.

Domingues and Boeger (2003).

######### Diplectanum squamatum

Taxon classificationAnimaliaDactylogyrideaDiplectanidae

Santos, Timi & Gibson, 2002

########## Type host.

*Cynoscion guatucupa* (Cuvier, 1830)

########## Infection site.

Gills.

########## Type locality.

Argentina, off Mar del Plata (38°08'S, 57°32'W).

########## Holotype.

CHIOC 34538 a.

########## Paratypes.

CHIOC 34538 b–d.

########## Remarks.

Other paratype deposited in the BMNH collection.

########## Reference.

Santos et al. (2002).

######## *Pseudorhabdosynochus* Yamaguti, 1958

######### Psedorhabdosynochus jeanloui

Taxon classificationAnimaliaDactylogyrideaDiplectanidae

Knoff, Cohen, Cárdenas, Cárdenas-Callirgos & Gomes, 2015

########## Type host.

*Paranthias colonus* (Valenciennes, 1846) (Osteichthyes: Serranidae).

########## Infection site.

Gill arches.

########## Type locality.

Peru, Lima, off Chorrillos (12°13'25"S, 76°43'49"W).

########## Holotype.

CHIOC 38016 a.

########## Paratypes.

CHIOC 38016 b–i.

########## Remarks.

Other paratype deposited in the MNHN collection.

########## Reference.

Knoff et al. (2015).

######### Pseudorhabdosynochus sulamericanus

Taxon classificationAnimaliaDactylogyrideaDiplectanidae

Santos, Buchmann & Gibson, 2000

########## Type host.

*Hyporthodus niveatus* (Valenciennes, 1828) [= *Epinephelus niveatus*] (Osteichthyes: Serranidae).

########## Infection site.

Gills.

########## Type locality.

Brazil, Rio de Janeiro State, Rio de Janeiro, off Cagarras Islands (23°02'S, 43°12'W).

########## Holotype.

CHIOC 33985 a.

########## Paratypes.

CHIOC 33985 b–i.

########## Remarks.

Other paratype deposited in the BMNH collection.

########## Reference.

Santos et al. (2000).

######## *Rhabdosynochus* Mizelle & Blatz, 1941

######### Rhabdosynochus hargisi

Taxon classificationAnimaliaDactylogyrideaDiplectanidae

Kritsky, Boeger & Robaldo, 2001

########## Type host.

*Centropomus undecimalis* (Bloch, 1792) (Osteichthyes: Centropomidae).

########## Infection site.

Gills.

########## Type locality.

Brazil, Pernambuco State, Itamaracá, earthen ponds (7°41'S, 34°53'W).

########## Paratypes.

CHIOC 34298 a–d.

########## Remarks.

Holotype deposited in the INPA collection. Other paratypes deposited in INPA, MPM, HWML and USNPC.

########## Reference.

Kritsky et al. (2001).

######### Rhabdosynochus hudsoni

Taxon classificationAnimaliaDactylogyrideaDiplectanidae

Kritsky, Boeger & Robaldo, 2001

########## Type host.

*Centropomus undecimalis*

########## Infection site.

Gills.

########## Type locality.

Brazil, Pernambuco State, Itamaracá, earthen ponds (7°41'S, 34°53'W).

########## Paratypes.

CHIOC 34297 a–d.

########## Remarks.

Holotype deposited in the INPA collection. Other paratypes deposited in INPA, MPM, HWML and USNPC.

########## Reference.

Kritsky et al. (2001).

######## *Spinomatrix* Boeger, Fehlauer & Marques, 2006

######### * Spinomatrix penteormos

Taxon classificationAnimaliaDactylogyrideaDiplectanidae

Boeger, Fehlauer & Marques, 2006

########## Type host.

*Pachyurus adspersus* Steindachner, 1879

########## Infection site.

Gills.

########## Type locality.

Brazil, Minas Gerais State, Vau-Açú, Piranga River, Cachoeirinha da Brecha downstream (20°32'S, 42°57'W).

########## Paratypes.

CHIOC 36502 a–f.

########## Remarks.

Holotype deposited in MZUSP. Other paratypes deposited in INPA, MZUSP, MNHN, HWML and USNPC.

########## Reference.

Boeger et al. (2006).

####### Gyrodactylidae Van Beneden & Hesse, 1863

######## *Diechodactylus* Vianna, Boeger & Silva-Souza, 2008

######### Diechodactylus joaberi

Taxon classificationAnimaliaGyrodactylideaGyrodactylidae

Vianna, Boeger & Silva-Souza, 2008

########## Type host.

*Gymnotus carapo* (Linnaeus, 1758) (Osteichthyes: Gymnotidae).

########## Infestation site.

Body surface.

########## Type locality.

Brazil, São Paulo State, São Carlos, Córrego do Feijão stream (22°06'10.7"S, 47°44'36.7"W).

########## Holotype.

CHIOC 36854 a.

########## Paratypes.

CHIOC 36854 b–e.

########## Remarks.

Other paratypes deposited in INPA and USNPC.

########## Reference.

Vianna et al. (2008).

######## *Gyrodactylus* Nordmann, 1832

######### Gyrodactylus anisopharynx

Taxon classificationAnimaliaGyrodactylideaGyrodactylidae

Popazoglo & Boeger, 2000

########## Type host.

*Corydoras paleatus* (Jenyns, 1842) (Osteichthyes: Callichthyidae).

########## Infestation site.

Body surface.

########## Type locality.

Brazil, Paraná State, Piraquara, Piraquara River (25°29'59"S, 49°02'40"W).

########## Holotype.

CHIOC 34215.

########## Paratypes.

CHIOC 34217 a–d, 34218 a–b, 34219, 34220, 34221 b, g–j, 34222.

########## Remarks.

Other paratypes deposited in IPCR, MNHN, HWML and USNPC. CHIOC 34216, 34221 a, c–f and 32223 were cited as paratypes in the original description, but they are paratypes of *Gyrodactylus corydori* Bueno-Silva & Boeger (2009).

########## References.

Popazoglo and Boeger (2000), Bueno-Silva and Boeger (2009).

######### Gyrodactylus carolinae

Taxon classificationAnimaliaGyrodactylideaGyrodactylidae

Boeger, Ferreira, Vianna & Patella, 2014

########## Type host.

*Characidium lanei*

########## Infestation site.

Body surface.

########## Type locality.

Brazil, Paraná State, Morretes, Marumbi River (25°29'27"S, 45°49'67"W).

########## Holotype.

CHIOC 37880.

########## Paratypes.

CHIOC 37875, 37876, 37877, 37878, 37879, 37900.

########## Remarks.

Other paratypes deposited in IPCAS, HWML and USNPC.

########## Reference.

Boeger et al. (2014).

######### Gyrodactylus corydori

Taxon classificationAnimaliaGyrodactylideaGyrodactylidae

Bueno-Silva & Boeger, 2009

########## Type host.

*Corydoras paleatus*

########## Infestation site.

Body surface.

########## Type locality.

Brazil, Paraná State, São José dos Pinhais, Miringuava River (25°38'06"S, 49°05'07"W).

########## Paratypes.

CHIOC 34216, 34221 a, c–f, 34223 a–b, 36911, 36912.

########## Remarks.

Paratypes from CHIOC collected in the Piraquara River (25°29'59"S, 49°02'40"W), Piraquara (Paraná State). Holotype deposited in MZUSP. Other paratypes deposited in MZUSP, MNHN, IPCR, HWML and USNPC.

########## Reference.

Bueno-Silva and Boeger (2009).

######### Gyrodactylus geophagensis

Taxon classificationAnimaliaGyrodactylideaGyrodactylidae

Boeger & Popazoglo, 1995

########## Type host.

*Geophagus brasiliensis*

########## Infestation site.

Body surface.

########## Type locality.

Brazil, Rio de Janeiro State, Itaguaí, Da Guarda River.

########## Holotype.

CHIOC 33144 a.

########## Paratypes.

CHIOC 33144 b–e

########## Remarks.

Other paratypes deposited in the collections of HWML and USNM.

########## Reference.

Boeger and Popazoglo (1995).

######### Gyrodactylus inesperatus

Taxon classificationAnimaliaGyrodactylideaGyrodactylidae

Boeger, Ferreira, Vianna & Patella, 2014

########## Type host.

*Characidium* sp.

########## Infestation site.

Body surface.

########## Type locality.

Brazil, São Paulo State, Ribeirão Bonito, Das Almas River (24°8'52"S, 48°20'52"W).

########## Holotype.

CHIOC 37869.

########## Paratypes.

CHIOC 37870, 37871, 37872, 37873, 37874.

########## Remarks.

Other paratypes deposited in IPCAS, HWML and USNPC.

########## Reference.

Boeger et al. (2014).

######### Gyrodactylus samirae

Taxon classificationAnimaliaGyrodactylideaGyrodactylidae

Popazoglo & Boeger, 2000

########## Type host.

*Corydoras ehrhardti* Steindachner, 1910

########## Infestation site.

Body surface.

########## Type locality.

Brazil, Paraná State, Piraquara, Piraquara River.

########## Holotype.

CHIOC 34226 a.

########## Paratypes.

CHIOC 34226 b, 34227, 34228, 34229 a–g.

########## Remarks.

Other paratypes deposited in IPCR, MNHN, HWML and USNPC.

########## Reference.

Popazoglo and Boeger (2000).

######### Gyrodactylus trairae

Taxon classificationAnimaliaGyrodactylideaGyrodactylidae

Boeger & Popazoglo, 1995

########## Type host.

Hoplias aff. malabaricus

########## Infestation site.

Body surface.

########## Type locality.

Brazil, Rio de Janeiro State, Nova Iguaçú, Guandu River.

########## Holotype.

CHIOC 33215 a.

########## Paratypes.

CHIOC 33215 b–i.

########## Remarks.

Other paratypes deposited in the collections of HWML and USNM.

########## Reference.

Boeger and Popazoglo (1995).

######## *Hiperoplestes* Boeger, Kritsky & Belmont-Jégu, 1994

######### * Hiperopletes malmbergi

Taxon classificationAnimaliaGyrodactylideaGyrodactylidae

Boeger, Kritsky & Belmont-Jégu, 1994

########## Type host.

*Rhineloricaria* sp. (Osteichthyes: Loricariidae).

########## Infestation site.

Body surface.

########## Type locality.

Brazil, Amazonas State, Manaus, Candiru stream.

########## Holotype.

CHIOC 33050 a.

########## Paratypes.

CHIOC 33050 b–l.

########## Remarks.

Other paratypes deposited in the collections of HWML and USNM.

########## Reference.

Boeger et al. (1994).

######## *Nothogyrodactylus* Kritsky & Boeger, 1991

######### Nothogyrodactylus amazonicus

Taxon classificationAnimaliaGyrodactylideaGyrodactylidae

Kritsky & Boeger, 1991

########## Type host.

*Ancistrus* sp. (Osteichthyes: Loricariidae).

########## Infestation site.

Skin.

########## Type locality.

Brazil, Amazonas State, Manaus, Bairro São Jorge.

########## Paratype.

CHIOC 32706.

########## Remarks.

Holotype deposited in the INPA collection. Other paratypes deposited in BMNH, HWML, USNM and ZIAC.

########## Reference.

Kritsky and Boeger (1991).

######### * Nothogyrodactylus clavatus

Taxon classificationAnimaliaGyrodactylideaGyrodactylidae

Kritsky & Boeger, 1991

########## Type host.

*Ancistrus* sp.

########## Infestation site.

Skin.

########## Type locality.

Brazil, Amazonas State, Manaus, Bairro São Jorge.

########## Paratype.

CHIOC 32705.

########## Remarks.

Holotype deposited in the INPA collection. Other paratypes deposited in BMNH, HWML, USNM and ZIAC.

########## Reference.

Kritsky and Boeger (1991).

######### Nothogyrodactylus plaesiophallus

Taxon classificationAnimaliaGyrodactylideaGyrodactylidae

Kritsky & Boeger, 1991

########## Type host.

*Ancistrus* sp.

########## Infestation site.

Skin.

########## Type locality.

Brazil, Amazonas State, Manaus, Bairro São Jorge.

########## Paratype.

CHIOC 32707.

########## Remarks.

Holotype deposited in the INPA collection. Other paratypes deposited in BMNH, HWML, USNM and ZIAC.

########## Reference.

Kritsky and Boeger (1991).

######## *Phanerothecium* Kritsky & Thatcher, 1977

######### Phanerothecium harrisi

Taxon classificationAnimaliaGyrodactylideaGyrodactylidae

Kritsky & Boeger, 1991

########## Type host.

*Hypostomus plecostomus* (Linnaeus, 1758) [= *Plecostomus plecostomus*] (Osteichthyes: Loricariidae).

########## Infestation site.

Skin.

########## Type locality.

Brazil, Amazonas State, Manaus, Bairro São Jorge.

########## Paratype.

CHIOC 32704.

########## Remarks.

Holotype deposited in the INPA collection. Other paratypes deposited in BMNH, HWML, USNM and ZIAC.

########## Reference.

Kritsky and Boeger (1991).

######### Phanerothecium spinatus

Taxon classificationAnimaliaGyrodactylideaGyrodactylidae

Boeger, Kritsky & Belmont-Jégu, 1994

########## Type host.

*Hypostomus punctatus* Valenciennes, 1840 (Osteichthyes: Loricariidae).

########## Infestation site.

Body surface.

########## Type locality.

Brazil, Rio de Janeiro State, Nova Iguaçú, Guandú River.

########## Holotype.

CHIOC 33051 a.

########## Paratypes.

CHIOC 33051 b–k.

########## Remarks.

Other paratypes deposited in the collections of HWML and USNM.

########## Reference.

Boeger et al. (1994).

######## *Polyclithrum* Rogers, 1967

######### Polyclithrum boegeri

Taxon classificationAnimaliaGyrodactylideaGyrodactylidae

Ernst, Whittington & Jones, 2000

########## Type host.

*Mugil liza* (= *Mugil platanus* Günther, 1880)

########## Infestation site.

Body washings.

########## Type locality.

Brazil, Rio de Janeiro State, Itaguaí, Da Guarda River.

########## Holotype.

CHIOC 33988 a.

########## Paratype.

CHIOC 33988 b.

########## Remarks.

Other paratypes deposited in the QM collection.

########## Reference.

Ernst et al. (2000).

####### Monocotylidae Taschenberg, 1879

######## *Heterocotyle* Scott, 1904

######### Heterocotyle sulamericana

Taxon classificationAnimaliaMonocotylideaMonocotylidae

Santos, Santos, Cunha & Chisholm, 2012

########## Type host.

*Dasyatis guttata* (Bloch & Schneider, 1801) (Osteichthyes: Dasyatidae).

########## Infection site.

Gills, between secondary lamellae.

########## Type locality.

Brazil, Rio de Janeiro State, off Angra dos Reis (23°00'24"S, 44°19'05"W).

########## Holotype.

CHIOC 37551 a.

########## Paratypes.

CHIOC 37551 b–d.

########## Remarks.

Collection numbers referred to as “3751” in the original description due to a mistake. Other paratypes deposited in AHC SAMA.

########## Reference.

Santos et al. (2012).

######## *Monocotyle* Taschenberg, 1878

######### Monocotyle guttatae

Taxon classificationAnimaliaMonocotylideaMonocotylidae

Santos, Santos & Gibson, 2006

########## Type host.

*Dasyatis guttata*

########## Infection site.

Gills.

########## Type locality.

Brazil, Rio de Janeiro State, off Angra dos Reis (23°00'24"S, 44°19'05"W).

########## Holotype.

CHIOC 36577.

########## Paratypes.

CHIOC 36578 a–c.

########## Remarks.

Other paratype deposited in the BMNH collection.

########## Reference.

Santos et al. (2006).

######## *Potamotrygonocotyle* Mayes, Brooks & Thorson, 1981

######### Potamotrygonocotyle aramasae

Taxon classificationAnimaliaMonocotylideaMonocotylidae

Domingues, Pancera & Marques, 2007

########## Type host.

*Paratrygon aiereba* (Müller & Henle, 1841) (Chondricthyes: Potamotrygonidae).

########## Infection site.

Gills.

########## Type locality.

Brazil, Amazonas State, Barcelos, Negro River (0°58'11"S, 62°55'13"W)

########## Paratypes.

CHIOC 36886, 36887 a–c.

########## Remarks.

Holotype deposited in MZUSP. Other paratypes deposited in INPA, MZUSP, IPCR, HWML and USNPC.

########## Reference.

Domingues et al. (2007).

######### Potamotrygonocotyle auriculocotyle

Taxon classificationAnimaliaMonocotylideaMonocotylidae

Domingues & Marques, 2011

########## Type host.

*Potamotrygon motoro* (Müller & Henle, 1841) (Chondricthyes: Potamotrygonidae).

########## Infection site.

Gills.

########## Type locality.

Brazil, Pará State, Cachoeira do Arari, Arari River, Urubu stream (1°00'36"S, 48°57'36"W).

########## Paratype.

CHIOC 37399.

########## Remarks.

Holotype deposited in MZUSP. Other paratypes deposited in INPA and MZUSP. Paratypes collected in a different site along the Urubu stream (0°59'58.2"S, 48°57'52.5594"W).

########## Reference.

Domingues and Marques (2011).

######### Potamotrygonocotyle chisholmae

Taxon classificationAnimaliaMonocotylideaMonocotylidae

Domingues & Marques, 2007

########## Type host.

*Potamotrygon motoro*

########## Infection site.

Gills.

########## Type locality.

Brazil, Mato Grosso do Sul State, Miranda, Salobra River (20°14'26"S, 56°22'42"W).

########## Paratypes.

CHIOC 36699 a–e.

########## Remarks.

Holotype deposited in MZUSP. Other paratypes deposited in INPA, MZUSP, HWML and USNPC.

########## Reference.

Domingues and Marques (2007).

######### Potamotrygonocotyle dromedarius

Taxon classificationAnimaliaMonocotylideaMonocotylidae

Domingues & Marques, 2007

########## Type host.

*Potamotrygon motoro*

########## Infection site.

Gills.

########## Type locality.

Brazil, Mato Grosso do Sul State, Miranda, Salobra River (20°14'26"S, 56°22'42"W).

########## Paratype.

CHIOC 36802.

########## Remarks.

Holotype deposited in MZUSP. Other paratypes deposited in INPA, MZUSP, HWML and USNPC.

########## Reference.

Domingues and Marques (2007).

######### Potamotrygonocotyle eurypotamoxenus

Taxon classificationAnimaliaMonocotylideaMonocotylidae

Domingues & Marques, 2007

########## Type host.

Potamotrygon cf. motoro

########## Infection site.

Gills.

########## Type locality.

Brazil, Mato Grosso do Sul State, Miranda, Salobra River (20°14'26"S, 56°22'42"W).

########## Paratype.

CHIOC 36803.

########## Remarks.

Holotype deposited in MZUSP. Other paratypes deposited in INPA, MZUSP, HWML and USNPC. Domingues and Marques (2011) considered this species to be a synonym of *Potamotrygon tsalickisi* Mayes, Brooks & Thorson, 1981.

########## References.

Domingues and Marques (2007), Domingues and Marques (2011).

######### Potamotrygonocotyle quadracotyle

Taxon classificationAnimaliaMonocotylideaMonocotylidae

Domingues, Pancera & Marques, 2007

########## Type host.

*Potamotrygon* sp.

########## Infection site.

Gills.

########## Type locality.

Brazil, Amazonas State, Barcelos, Negro River (0°58'11"S, 62°55'13"W).

########## Paratypes.

CHIOC 36883 a–d.

########## Remarks.

Holotype deposited in MZUSP. Other paratypes deposited in INPA, MZUSP, IPCR, HWML and USNPC.

########## Reference.

Domingues et al. (2007).

######### Potamotrygonocotyle rionegrense

Taxon classificationAnimaliaMonocotylideaMonocotylidae

Domingues, Pancera & Marques, 2007

########## Type host.

*Potamotrygon* sp.

########## Infection site.

Gills.

########## Type locality.

Brazil, Amazonas State, Barcelos, Negro River (0°58'11"S, 62°55'13"W).

########## Paratypes.

CHIOC 36885 a–d.

########## Remarks.

Holotype deposited in MZUSP. Other paratypes deposited in INPA, MZUSP, IPCR, HWML and USNPC.

########## Reference.

Domingues et al. (2007).

######### Potamotrygonocotyle septemcotyle

Taxon classificationAnimaliaMonocotylideaMonocotylidae

Domingues & Marques, 2011

########## Type host.

*Potamotrygon scobina* Garman, 1913

########## Infection site.

Gills.

########## Type locality.

Brazil, Pará State, Colares, Marajó Bay, Tocantins River (0°55'45"S, 48°17'29"W).

########## Paratypes.

CHIOC 37397 a–b.

########## Remarks.

Holotype deposited in MZUSP. Other paratypes deposited in INPA, MZUSP and USNPC.

########## Reference.

Domingues and Marques (2011).

######### Potamotrygonocotyle tatianae

Taxon classificationAnimaliaMonocotylideaMonocotylidae

Domingues & Marques, 2011

########## Type host.

*Paratrygon* sp.

########## Infection site.

Gills.

########## Type locality.

Brazil, Amazonas State, Benjamin Constant, Yavari River (4°18'15.1194"S, 70°4'19.56"W).

########## Paratype.

CHIOC 37404.

########## Remarks.

Holotype deposited in MZUSP. Other paratypes deposited in INPA, MZUSP, HMWL and USNPC. Paratypes collected in a different site along the Yavari River (4°18'25"S, 70°4'31"W).

########## Reference.

Domingues and Marques (2011).

######### Potamotrygonocotyle tocantinsense

Taxon classificationAnimaliaMonocotylideaMonocotylidae

Domingues & Marques, 2011

########## Type host.

Potamotrygon cf. scobina

########## Infection site.

Gills.

########## Type locality.

Brazil, Tocantins State, Ipueiras, Tocantins River (11°18'36"S, 48°27'36"W).

########## Paratypes.

CHIOC 37405 a–c.

########## Remarks.

Holotype deposited in MZUSP. Other paratypes deposited in INPA, MZUSP and USNPC. Paratypes collected from a different site along the Tocantins River (11°15'48.52"S, 48°26'56.79"W).

########## Reference.

Domingues and Marques (2011).

######### Potamotrygonocotyle umbella

Taxon classificationAnimaliaMonocotylideaMonocotylidae

Domingues, Pancera & Marques, 2007

########## Type host.

*Potamotrygon* sp.

########## Infection site.

Gills.

########## Type locality.

Brazil, Amazonas State, Barcelos, Negro River (0°58'11"S, 62°55'13"W).

########## Paratypes.

CHIOC 36884 a–b.

########## Remarks.

Holotype deposited in MZUSP. Other paratypes deposited in INPA, MZUSP, IPCR, HWML and USNPC.

########## Reference.

Domingues et al. (2007).

######### Potamotrygonocotyle uruguayensis

Taxon classificationAnimaliaMonocotylideaMonocotylidae

Domingues & Marques, 2007

########## Type host.

*Potamotrygon brachyura* (Günther, 1880)

########## Infection site.

Gills.

########## Type locality.

Brazil, Rio Grande do Sul State, Porto Xavier, Uruguay River (27°54'00"S, 55°08'00"W).

########## Paratypes.

CHIOC 36807 a–d.

########## Remarks.

Domingues and Marques (2011) considered this species to be a synonym of *Potamotrygonocotyle chisholmae*. Holotype deposited in MZUSP. Other paratypes deposited in INPA, MZUSP, HWML and USNPC.

########## References.

Domingues and Marques (2007), Domingues and Marques (2011).

###### Subclass Polystomatoinea Lebedev, 1986

####### Polystomatidae Gamble, 1896

######## *Neopolystoma* Price, 1939

######### Neopolystoma fentoni

Taxon classificationAnimaliaPolystomatideaPolystomatidae

Platt, 2000

########## Type host.

*Cryptochelys leucostoma* (Duméril & Bibron, 1851) (Testudines: Kinosternidae)

########## Infection site.

Conjunctival sac.

########## Type locality.

Costa Rica, Guanacaste, Santa Rosa, Guanacaste Conservation Area, Quebrada Costa Rica (10°49.666'N, 85°38.216'W).

########## Paratypes.

CHIOC 34292, 34293 a–b, 34294.

########## Remarks.

CHIOC 34292 and 34293 a–b were collected from *Rhinoclemmys pulcherrima* (Gray, 1856) (Testudines: Geoemydidae) in Quebrada El Duende (10°50.236'N, 85°36.724'W), Guanacaste Conservation Area. Holotype and other paratypes deposited in USNPC.

########## Reference.

Platt (2000).

######## *Polystomoides* Ward, 1917

######### Polystomoides brasiliensis

Taxon classificationAnimaliaPolystomatideaPolystomatidae

Vieira, Novelli, Sousa & SouzaLima, 2008

########## Type host.

*Hydromedusa maximiliani* (Mikan, 1825) (Testudines: Chelidae).

########## Infection site.

Buccal and pharyngeal cavities.

########## Type locality.

Brazil, Minas Gerais State, Juiz de Fora, lake of Mariano Procópio Museum (21°41'20"S, 43°20'40"W).

########## Holotype.

CHIOC 36902.

########## Paratypes.

CHIOC 36903 a–d.

########## Reference.

Vieira et al. (2008).

###### Subclass Oligochoinea Bychowsky, 1937

####### Allodiscocotylidae Tripathi, 1959

######## *Metacamopiella* Kohn, Santos & Lebedev, 1996

######### * Metacamopiella euzeti

Taxon classificationAnimaliaMazocraeideaAllodiscocotylidae

Kohn, Santos & Lebedev, 1996

########## Type host.

*Trachinotus carolinus* (Linnaeus, 1766) (Osteichthyes: Carangidae).

########## Infection site.

Gills.

########## Type locality.

Brazil, Rio de Janeiro State, Guanabara Bay (23°48'S, 43°10'W).

########## Holotype.

CHIOC 33059.

########## Remarks.

Paratypes deposited in PC–IBPRAS.

########## Reference.

Kohn et al. (1996).

######## Metacamopia oligoplites

Taxon classificationAnimaliaMazocraeideaAllodiscocotylidae

Takemoto, Amato & Luque, 1996

######### Type host.

*Oligoplites palometa* (Cuvier, 1833) (Osteichthyes: Carangidae).

######### Infection site.

Gills.

######### Type locality.

Brazil, Rio de Janeiro State, Itacuruça, Sepetiba Bay (22°51'S, 43°56'W).

######### Holotype.

CHIOC 33623 a.

######### Paratypes.

CHIOC 33623 b–c, 33624, 33625.

######### Remarks.

Other paratypes deposited in USNPC.

######### Reference.

Takemoto et al. (1996).

####### Anoplodiscidae Tagliani, 1912

######## *Anoplodiscus* Sonsino, 1890

######### Anoplodiscus longivaginatus

Taxon classificationAnimaliaGyrodactylideaAnoplodiscidae

Paraguassú, Luque & Alves, 2002

########## Type host.

*Pagrus pagrus* (Linnaeus, 1758) (Osteichthyes: Sparidae).

########## Infection site.

Mouth and gills.

########## Type locality.

Brazil, coastal zone of Rio de Janeiro State (21–23°S, 41–45°W).

########## Holotype.

CHIOC 34905.

########## Paratypes.

CHIOC 34906 a–d, 34907.

########## Remarks.

Other paratypes deposited in MPM and USNPC.

########## Reference.

Paraguassú et al. (2002).

####### Diclidophoridae Cerfontaine, 1895

######## *Absonifibula* Lawler & Overstreet, 1976

######### Absonifibula estuarina

Taxon classificationAnimaliaMazocraeideaDiclidophoridae

Santos & Timi, 2009

########## Type host.

*Cynoscion guatucupa*

########## Infection site.

Gills.

########## Type locality.

Argentina, Buenos Aires Province, Mar del Plata (38°08'S 57°32'W).

########## Paratypes.

CHIOC 37205 a–d.

########## Remarks.

Holotype and other paratypes deposited in CHMLP.

########## Reference.

Santos and Timi (2009).

######## *Choricotyle* van Beneden & Hesse, 1863

######### Choricotyle brasiliensis

Taxon classificationAnimaliaMazocraeideaDiclidophoridae

Luque, Amato & Takemoto, 1993

########## Type host.

*Orthopristis ruber* (Cuvier, 1830) (Osteichthyes: Haemulidae).

########## Infection site.

Gills.

########## Type locality.

Brazil, Rio de Janeiro State, Sepetiba Bay (22°51'S, 43°56'W).

########## Paratypes.

CHIOC 33105, 33106.

########## Remarks.

Holotype and other paratypes deposited in the USNM collection.

########## Reference.

Luque et al. (1993a).

######### Choricotyle orthopristis

Taxon classificationAnimaliaMazocraeideaDiclidophoridae

Luque, Amato & Takemoto, 1993

########## Type host.

*Orthopristis ruber*

########## Infection site.

Gills.

########## Type locality.

Brazil, Rio de Janeiro State, Sepetiba Bay (22°51'S, 43°56'W).

########## Paratypes.

CHIOC 33108 a–b.

########## Remarks.

One of the collection numbers is referred to as “38108b” in the original description due to a mistake. Holotype and other paratypes deposited in the USNM collection.

########## Reference.

Luque et al. (1993a).

######### Choricotyle rohdei

Taxon classificationAnimaliaMazocraeideaDiclidophoridae

Cohen, Cárdenas, Fernandes & Kohn, 2011

########## Type host.

*Ctenosciaena gracilicirrhus* (Metzelaar, 1919) (Osteichthyes: Sciaenidae).

########## Infection site.

Gill lamellae.

########## Type locality.

Brazil, Rio de Janeiro State, Angra dos Reis (23°00'24"S, 44°19'05"W).

########## Holotype.

CHIOC 37473 a.

########## Paratypes.

CHIOC 37473 b, 37474 a–b, 37475, 37476 a–b, 37477 a–b, 37478, 37479 a–b, 37480, 37481, 37482, 37483 a–c, 37484 a–b, 37485, 37486, 37487, 37488 a–b, 37489 a–c, 37490 a–c, 37491.

########## Reference.

Cohen et al. (2011).

####### Heteraxinidae Unnithan, 1957

######## *Probursata* Bravo-Hollis, 1984

######### Probursata brasiliensis

Taxon classificationAnimaliaMazocraeideaHeteraxinidae

Takemoto, Amato & Luque, 1993

########## Type host.

*Oligoplites palometa*

########## Infection site.

Gill filaments.

########## Type locality.

Brazil, Rio de Janeiro State, Sepetiba Bay, Itacuruçá (22°61'S, 43°56'W).

########## Holotype.

CHIOC 33069.

########## Paratypes.

CHIOC 33053 a–b, 33054, 33055, 33056, 33057.

########## Remarks.

Other paratypes deposited in the USNM collection.

########## Reference.

Takemoto et al. (1993).

####### Macrovalvitrematidae Yamaguti, 1963

######## *Pseudotagia* Yamaguti, 1963

######### Pseudotagia rubri

Taxon classificationAnimaliaMazocraeideaMacrovalvitrematidae

Luque, Amato & Takemoto, 1993b

########## Type host.

*Orthopristis ruber*

########## Infection site.

Gills.

########## Type locality.

Brazil, Rio de Janeiro State, Sepetiba Bay, Itacuruçá (22°51'S, 43°56'W).

########## Paratypes.

CHIOC 33100, 33101, 33102.

########## Remarks.

Holotype and other paratypes deposited in the USNM collection.

########## Reference.

Luque et al. (1993b).

####### Mazocraeidae Price, 1936

######## *Cribomazocraes* Mamaevm, 1981

######### Cribomazocraes travassosi

Taxon classificationAnimaliaMazocraeideaMazocraeidae

Santos & Kohn, 1992

########## Type host.

*Harengula clupeola* (Cuvier, 1829) (Osteichthyes: Clupeidae).

########## Infection site.

Gills.

########## Type locality.

Brazil, Rio de Janeiro State, Rio de Janeiro, Ilha do Governador.

########## Holotype.

CHIOC 32758.

########## Remarks.

No paratype deposited, only vouchers.

########## Reference.

Santos and Kohn (1992).

####### Microcotylidae Taschenberg, 1879

######## *Microcotyle* van Beneden & Hesse, 1863

######### Microcotyle pseudopercis

Taxon classificationAnimaliaMazocraeideaMicrocotylidae

Amato & Cezar, 1994

########## Type host.

*Pseudopercis numida* Miranda-Ribeiro, 1903 (Osteichthyes: Pinguipedidae).

########## Infection site.

Gill filaments.

########## Type locality.

Brazil, coast of Rio de Janeiro State (21–23°S, 41–45°W).

########## Paratypes.

CHIOC 33290 a–b.

########## Remarks.

Holotype and other paratype deposited in the USNM collection.

########## Reference.

Amato and Cezar (1994).

######## *Paranaella* Kohn, Baptista-Farias & Cohen, 2000

######### * Paranaella luquei

Taxon classificationAnimaliaMazocraeideaMicrocotylidae

Kohn, Baptista-Farias & Cohen, 2000

########## Type host.

*Hypostomus* sp.

########## Infection site.

Gills.

########## Type locality.

Brazil, Paraná State, Paraná River, reservoir of the Itaipu Hydroelectric Power Station.

########## Holotype.

CHIOC 33954 a.

########## Paratypes.

CHIOC 33954 b–h.

########## Remarks.

There is no paratype “i”, as informed in the original description. That was a mistake.

########## Reference.

Kohn et al. (2000).

####### Plectanocotylidae Poche, 1926

######## *Octoplectanocotyla* Yamaguti, 1937

######### Octoplectanocotyla travassosi

Taxon classificationAnimaliaMazocraeideaPlectanocotylidae

Carvalho & Luque, 2012

########## Type host.

*Trichiurus lepturus*

########## Infection site.

Gills.

########## Type locality.

Brazil, Rio de Janeiro State, Guanabara Bay (23°1'52"S, 43°11'56"W).

########## Holotype.

CHIOC 37386.

########## Paratypes.

CHIOC 37387 a–b, 37388 a–b.

########## Reference.

Carvalho and Luque (2012).
